# Screening of Natural Compounds as P-Glycoprotein Inhibitors against Multidrug Resistance

**DOI:** 10.3390/biomedicines9040357

**Published:** 2021-03-30

**Authors:** Sérgio M. Marques, Lucie Šupolíková, Lenka Molčanová, Karel Šmejkal, David Bednar, Iva Slaninová

**Affiliations:** 1 Loschmidt Laboratories, Department of Experimental Biology and Research Centre for Toxic Compounds in the Environment RECETOX, Faculty of Science, Masaryk University, Kamenice 5/C13, 625-00 Brno, Czech Republic; smar96@gmail.com; 2 International Clinical Research Center, St. Anne’s University Hospital Brno, Pekarska 53, 656-91 Brno, Czech Republic; 3 Department of Biology, Faculty of Medicine, Masaryk University, Kamenice 5/A6, 625-00 Brno, Czech Republic; supolikova@med.muni.cz; 4 Department of Natural Drugs, Faculty of Pharmacy, Masaryk University, Palackého 1946/1, 612-00 Brno, Czech Republic; lenka.molcanova1993@gmail.com (L.M.); karel.mejkal@post.cz (K.Š.)

**Keywords:** flavonoids, molecular dynamics, molecular docking, multidrug resistance, natural compounds, P-glycoprotein, structure-based virtual screening

## Abstract

Multidrug resistance (MDR) is a common problem when fighting cancer with chemotherapy. P-glycoprotein (P-gp, or MDR1) is an active pump responsible for the efflux of xenobiotics out of the cell, including anti-cancer drugs. It is a validated target against MDR. No crystal structure of the human P-gp is available to date, and only recently several cryo-EM structures have been solved. In this paper, we present a comprehensive computational approach that includes constructing the full-length three-dimensional structure of the human P-gp and its refinement using molecular dynamics. We assessed its flexibility and conformational diversity, compiling a dynamical ensemble that was used to dock a set of lignan compounds, previously reported as active P-gp inhibitors, and disclose their binding modes. Based on the statistical analysis of the docking results, we selected a system for performing the structure-based virtual screening of new potential P-gp inhibitors. We tested the method on a library of 87 natural flavonoids described in the literature, and 10 of those were experimentally assayed. The results reproduced the theoretical predictions only partially due to various possible factors. However, at least two of the predicted natural flavonoids were demonstrated to be effective P-gp inhibitors. They were able to increase the accumulation of doxorubicin inside the human promyelocytic leukemia HL60/MDR cells overexpressing P-gp and potentiate the antiproliferative activity of this anti-cancer drug.

## 1. Introduction

Multidrug resistance (MDR) is the common cause of therapeutic failure, not only in cancer but also in other diseases. One of the mechanisms lying behind the MDR is the efflux of drugs from tumor cells through the ATP binding cassette (ABC) transporters, which act as integral membrane pumps. The ABC transporters are classified into seven subfamilies (ABCA-ABCG) based on their sequence homology and domain organization [1].

The most typical ABC transporter is P-glycoprotein (P-gp; 170 kDa), coded by the *ABCB1* (*MDR1*) gene, which is often over-expressed in the cancer cells of tumors treated by anti-cancer drugs. It has been proposed to operate as a “hydrophobic vacuum cleaner,” expelling non-polar compounds from the cytosolic side of the membrane bilayer to the exterior [2,3,4]. Other important efflux transporters responsible for MDR are proteins encoded by the *ABCC1–6* genes (multidrug resistance-related proteins 1–6; MRP1–6) and the *ABCG2* gene (breast cancer resistant protein; BCRP) [5]. ABC transporters consist of minimally four domains: two nucleotide-binding domains (NBDs) with highly conserved sequence motifs and two transmembrane domains. The two NBDs contain conserved motifs such as Walker A, Walker B, ABC signature, a glutamine loop (Q-loop), and a switch motif. The activity of ABC transporters can be regulated by other domains that can bind to the NBDs or to the transmembrane domains [6,7]. The P-gp structure is formed by two pseudo-symmetrical halves with domain-swapping features. It includes at least two positively cooperative sites for drug binding, with the H site preferring Hoechst 33342 to rhodamine 123, and the R site preferring rhodamine 123 to Hoechst 33342. Binding to one of these sites has been shown to stimulate the binding to the other and the respective transport activity [8]. Other binding sites, namely a modulator-binding site (M site), have been reported and assigned in the P-gp structure [9]. The mechanisms of transport of substrates and drug modulation of P-gp have been extensively studied, both by experimental and computational methods [10,11,12,13]. Although the cycle is still not fully characterized, it is generally accepted that ATP binding to the NBDs triggers major conformational changes. The inward-facing conformation of P-gp, containing the substrates (drugs) bound in the transmembrane binding pocket, is converted into an outward-facing conformation that can release the substrates to the extracellular environment. The hydrolysis of ATP, enabled by the dimerization of the NBDs that led to the outward-facing conformation in the first place, restores the initial inward-facing state and closes the cycle [13,14].

A promising method for overcoming MDR based on an over-activation of these membrane pumps is the co-administration anti-cancer drugs with inhibitors of those pumps. Three generations of MDR inhibitors have been introduced in the clinical trials during the last decades. Unfortunately, their toxicity in the doses required for blocking ABC transporters disabled their use in the clinical practice [7]. Finding new non-toxic membrane pump inhibitors can help solve this problem. Natural products occupy a fundamental position among anti-cancer drugs, as more than 70% of all anti-cancer drugs currently on the market were derived from, or inspired by, natural products [15]. Non-toxic inhibitors or modulators originating from natural sources are sometimes referred to as “fourth generation inhibitors” [1]. Fumitremorgin C, a mycotoxin isolated from *Aspergillus fumigatus*, is an example of a natural product inhibiting drug efflux. It was the first reported inhibitor of BCRP. Its tetracyclic analogs (Ko132, Ko134, and Ko143) have even greater potential to inhibit BCRP with minimal toxicity.

Several research groups have described the ability of natural products, such as lignans and flavonoids, to inhibit P-gp-, MRP1-, and BCRP-mediated efflux and restore drug sensitivity in MDR cancer cells [1]. Flavonoids, a large group of polyphenolic compounds found in medicinal plants, vegetables, fruits, and beverages such as tea and wine, are an integral component of our everyday diet. In plants, they can be found as aglycones, but more often, they occur in the form of glycosides. Flavonoids can be classified as flavonols, flavones, isoflavones, flavanols, flavanones, and chalcones [16]. Flavonoids have a wide variety of biological activities, including anti-oxidant, anti-inflammatory, and anti-cancer. The proposed mechanisms for the anti-cancer effects include their anti-oxidant activities, their effects on signal transduction pathways involved in cell proliferation and angiogenesis, and their ability to modulate the activity of enzymes required for metabolic activation of procarcinogens and the detoxification of carcinogens [16]. Flavopiridol, a synthetic flavone currently in clinical trials as an antineoplastic agent, is a potent inhibitor of several kinases, including CDK2 and CDK4 [17].

In vitro studies [18] have revealed that flavonoids mostly modulate ABC drug transporters by competitively binding to their substrate-binding sites. However, some flavonoids bind to the NBDs, affecting the ATP binding or hydrolysis, or alter the surface expression level of ABC transporters [1]. Boumendjel et al. investigated the structure–activity relationships of flavonoids as potential MDR modulators. They concluded that flavonoids are bi-functionally able to partly overlap the ATP-binding site and a vicinal hydrophobic region interacting with steroids within a cytosolic domain of P-gp. They propose that flavonoids reveal the binding affinity toward NBD2 of P-gp through their ability to mimic the adenine moiety of ATP, which indicates that the flavonoid overlaps, at least partly, the nucleotide-binding site [19].

A particular group of flavonoids is represented by prenylated flavonoids, commonly possessing cytotoxic, antibacterial, and anti-inflammatory properties [20]. The prenylation is generally seen as the introduction of C5 (prenyl) or C10 (geranyl) moieties to different positions of the flavonoid skeleton. The side chain can be further modified. Prenylation of the flavonoid skeleton increases lipophilicity, which may enable these compounds to cross the cell membrane. Furthermore, the lipophilic side chain increases the chance of their interaction with various proteins, including those regulating cellular signaling. *Paulownia tomentosa* is an example of a plant source rich in such compounds, based on the combination of a flavanone skeleton with a geranyl at position C6 [21,22]. The cytotoxic properties of prenylated flavonoids were previously reviewed [23]. However, their activity against MDR was not systematically studied. We have previously demonstrated that a set of lignans (natural phenolic compounds), isolated from the magnolia-vine (*Schisandra chinensis* (Turcz.) Baill.), potentiate the cytotoxic effect of the anti-cancer drug doxorubicin, by increasing its accumulation inside the multidrug-resistant leukemia cells overproducing P-gp (HL60/MDR) [24].

To date, no crystal structure of the human P-gp has been solved. Hence, most of the modeling studies with P-gp modulators have used the mouse variant of that protein, which is often considered a good surrogate for the human variant due to a high homology of 87%. Other studies have used homology models of the human P-gp, some of which have suggested different properties than the mouse homolog arising from the sequence variation [25,26]. One of the main issues is related to the high flexibility of P-gp and the large conformational variability among the known crystal structures and models, which may lead to inconsistent results. Molecular dynamics (MD) simulations have been used to incorporate flexibility in the study of potential inhibitors or substrates and improve prediction accuracy. However, some caution is needed when interpreting the results [25,27]. Many modeling and virtual screening studies aiming to find P-gp modulators have been performed with different approaches and strategies [28,29,30], including several focused on natural compounds [31,32].

In this paper, we report a comprehensive approach that includes: (1) prediction of the three-dimensional structure of the human P-gp by molecular threading and its refinement with MD simulations; (2) docking of our previously reported inhibitors (lignans) to construct a general structure-based virtual screening methodology; (3) virtual screening of a library of 87 natural compounds (flavonoids); (4) experimental in vitro screening of the top-ranked available molecules for their validation as P-gp inhibitors on cancer cells overexpressing P-gp.

## 2. Materials and Methods

### 2.1. Molecular Threading

The 3D structure of the human P-gp (P-gp; GenBank code: AAA59575.1) [33,34] was predicted from the peptide, provided as the FASTA sequence, by molecular threading with the I-TASSER server [35,36] using the default settings. The Structural Analysis and Verification Server (SAVES v4.0) (https://servicesn.mbi.ucla.edu/SAVES/, accessed on 15 January 2021) and the Swiss-Model Structure Assessment server (https://swissmodel.expasy.org/assess, accessed on 15 January 2021) [37,38] were used to assess the stereo-chemical quality of these structures, by providing several parameters: C-score [35], TM-score [35,39], PROVE [40], ERRAT [41], VERIFY3D [42], QMEAN [43], MolProbity score [44], and Ramachandran plots [45]. The resulting models were also aligned with a crystal structure of the mouse P-gp (PDB ID: 4M1M) using PyMOL 2.3.2 [46] to assess the root-mean-square deviation (RMSD) of the C_α_ atoms. Based on these parameters, the top-ranked model 1 from I-TASSER was selected for further refinement and analyses.

### 2.2. Molecular Dynamics

To improve the structural quality and diversity of the predicted human P-gp, model 1, obtained from I-TASSER, was submitted to molecular dynamics simulations. The hydrogen atoms were calculated using the H++ server [47], with an implicit solvent at pH 7.4, 0.1 M salinity, with an internal dielectric constant of 10 and external of 80. The tLEaP program of AmberTools 14 [48] was then used to prepare the topology and coordinates files for performing the MD simulation. The system was neutralized by adding 17 Cl^−^ ions, and it was solvated with an octagonal box of TIP3P water molecules [49] with the edges at least 10 Å away from the protein atoms.

The equilibration and production MD simulations were carried out with the PMEMD.CUDA [50,51] module of AMBER 14, using the force field ff14SB [52]. This force field is currently one of the primary AMBER reference models for proteins, and it is recommended to be used in combination with the TIP3P water model (https://ambermd.org/AmberModels.php, accessed on 24 January 2021). In total, five minimization steps and 12 steps of equilibration dynamics were performed prior to the production MD. The first four minimization steps, composed of 2500 cycles of steepest descent followed by 7500 cycles of conjugate gradient, were performed as follows: (i) in the first one, all the atoms of the protein and ligand were restrained with 500 kcal∙mol^−1^∙Å^−2^ harmonic force constant; (ii) in the following ones, only the backbone atoms of the protein and heavy atoms of the ligand were restrained, respectively, with 500, 125, and 25 kcal∙mol^−1^∙Å^−2^ force constant. A fifth minimization step, composed of 5000 cycles of steepest descent and 15,000 cycles of the conjugate gradient, was performed without restraints. The subsequent MD simulations employed periodic boundary conditions, the particle mesh Ewald method was used for the treatment of the long-range interactions beyond the 10 Å cutoff [53], the SHAKE algorithm [54] was used to constrain the bonds involving the hydrogen atoms, and the Langevin thermostat was used with a collision frequency of 1.0 ps^−1^ and a time step of 2 fs. The energy and coordinates were saved every 2 ps. Equilibration dynamics were performed in 12 steps: (i) 20 ps of gradual heating from 0 to 310 K, under constant volume, restraining the protein atoms and ligand with 200 kcal∙mol^−1^∙Å^2^ harmonic force constant; (ii) 10 MDs of 400 ps each, at constant pressure (1 bar) and constant temperature (310 K), with gradually decreasing restraints on the backbone atoms of the protein and heavy atoms of the ligand with harmonic force constants of 150, 100, 75, 50, 25, 15, 10, 5, 1, and 0.5 kcal∙mol^−1^∙Å^−2^; (iii) 400 ps of MD at the same conditions as the previous ones, but with restraints of 0.5 kcal/mol^−1^∙Å^−2^ applied only on the backbone atoms of the transmembrane residues, as predicted by the PPM server [55]: residues 45–73, 75–76, 114–136, 188–232, 293–317, 329–352, 710–736, 753–777, 831–879, 935–959, and 973–993. The production MDs were run for 500 ns using the same settings employed in the last equilibration step. The trajectories were analyzed using the cpptraj [56] module of AmberTools 14, and visualized using PyMOL 2.3.2 [46] and VMD 1.9.1 [57].

The trajectory was clustered using *cpptraj*, with a distance-based metric of the mass-weighted RMSD of the residues located in the extended binding site in the transmembrane regions: residues 1–10, 44–74, 113–138, 187–234, 294–318, 330–353, 711–737, 751–775, 833–877, 934–961, and 972–995 (all the heavy atoms were included). The hierarchical agglomerative clustering algorithm was used with average-linkage, a cutoff for minimum distance between clusters (epsilon) of 1.5 Å, sieve 4, and a minimum of 10 clusters. The centroid structures of the clusters were saved and used in the subsequent docking analyses. These structures were analyzed with the Swiss-Model and SAVES servers for quality assessment.

### 2.3. Molecular Docking

The three-dimensional structures of all the ligands were prepared in Avogadro [58] and then minimized using the UFF force field [59] and the steepest descent algorithm. The *antechamber* module of AmberTools 14 was employed to calculate the partial charges of the ligands using the semi-empirical AM1-BCC function [60,61]. The receptor structures included: (i) the initial homology model 1, (ii) the structure after the equilibration MD, (iii) the 10 clusters’ representative structures, (iv) two crystal structures of the mouse P-gp (PDB IDs: 3G60 and 4M1M), and (v) the three available cryo-EM structures of the human P-gp (PDB IDs: 6C0V, 6QEE, and 6QEX). The mouse variants and cryo-EM structures were prepared by removing the B chains, when existing, co-crystallization ions and ligands, and the hydrogen atoms were added with the *reduce* program of AmberTools 14 using dynamic optimization of their position (*-build -nuclear* options). All structures were aligned prior to the docking using PyMOL 2.3.2 [46].

The input files of the ligands and receptors, in MOL2 and PDB formats, respectively, were converted to the AutoDock Vina-compatible format PDBQT MGLTools [62], maintaining the previously calculated atomic charges of the ligands. The ligand-binding site of the human P-gp, as identified by I-TASSER (residues 69, 72, 336, 40, 343, 725, 728, 732, 953, 975, 978, 979, 983, and 986), was used to define the region of interest for the molecular docking performed by AutoDock Vina [63]. This region was represented by a cubic box of 40 × 40 × 40 Å centered at the center of mass of the C_α_ atoms of those residues. Such a box was sufficiently large to include the H, M, and R binding sites. The exhaustiveness parameter was set to 100 (the default is 8), and the maximum number of binding modes (20) was saved. The docking poses obtained from AutoDock Vina were re-scored by the SMINA [64], NNScore [65], and the RF-Score-VS [66] scoring functions. The docked binding modes were visualized using PyMOL 2.3.2 [46].

### 2.4. Flavonoid Compounds

Tomentone (**1**), diplacone (**2**), mimulone (**3**), 5,7-dihydroxy-6-geranylchromone (**4**), tomentodiplacone M (**5**), tomentodiplacone L (**6**), tomentodiplacone N (**7**), 3′-*O*-methyldiplacol (**8**), 3′-*O*-methyl-5′-methoxydiplacol (**9**), and 3′-*O*-methyl-5′-methoxydiplacone (**10**) were obtained by the isolation procedures described in previous works (**1**–**3**, **5**–**8**, **10** [67], **4** [68], and **9** [69]). Briefly, the fruits of *P. tomentosa* (Paulowniaceae) were extracted with ethanol, further fractionated by liquid-liquid extraction to several portions, and the chosen portions were subsequently separated in several steps using column chromatography, flash chromatography, semipreparative RP-HPLC, or preparative TLC. The purity of the isolated compounds was evaluated using HPLC-DAD analysis and exceeded 95% in all cases. Structural elucidation was performed using UV and IR analysis, mass spectrometry, 1D and 2D NMR spectroscopy, and the absolute configurations were determined using circular dichroism in combination with NMR [67,68,69].

### 2.5. Cell Lines and Cultivation Conditions

The multidrug-resistant sub-line of human promyelocytic leukemia cells HL60/MDR overexpressing P-gp derived from parental HL60 cells after treatment with doxorubicin was obtained from Prof. B. Sarkadi (Semmelweis University of Medicine, National Blood Centre, Membrane Research Group, Hungarian Academy of Science, Budapest, Hungary). The cells were cultivated in RPMI 1640 medium supplemented with 2 mM glutamine and 10% fetal calf serum, 100 IU/mL penicillin, and 100 µg/mL streptomycin (PAA Laboratories, Austria) at 37 °C under 5% CO_2_ in a high-humidity atmosphere and subcultured three times a week. The characterization of the cells, the evidence of P-gp overexpression by immunodetection of P-gp, and RT-PCR detection of mRNA of P-gp were done as reported in a previous study [24].

### 2.6. Drug Accumulation Assay

A method described previously [24] was used to measure the fluorescent substrate doxorubicin content in the cells by flow cytometry. Briefly, the HL60/MDR cells (2 × 10^6^ per mL) were pre-incubated for 15 min in a medium containing either the flavonoids **1–10** (10 and 20 µM) or verapamil (20 µM), a first-generation inhibitor used as a positive control, and then incubated with doxorubicin (10 µM) for an additional 60 min. After washing with PBS, the intracellular content of doxorubicin was determined using a Cytomics FC 500 flow cytometer (Beckman Coulter, Inc., Carlsbad, CA, USA; an excitation wavelength of 488 nm and an emission wavelength of 575 nm; FL2). A minimum of 10,000 cells was counted for each sample. The results were expressed as the percentage ratio of the mean of fluorescence of doxorubicin in the presence of a modulator to the mean of fluorescence of doxorubicin without a modulator.

### 2.7. Cell Viability Assay

The cell viability assay was based on the exclusion of propidium iodide (PI; Sigma-Aldrich, St. Louis, MO, USA) by the intact viable cells. The cells (7 × 10^4^ cells/mL) were plated in 12-well tissue culture test plates (Orange Scientific, Braine-I’Alleud, Belgium) and treated with test compounds at a concentration of 20 µM in combination with doxorubicin at a concentration of 1 µM. After 48 h of incubation, PI was added, and the percentage of dead (PI-positive) cells was detected using a Cytomics FC 500 flow cytometry system (Beckman Coulter, Inc., Brea, CA, USA) in channel FL3 (emission at 620 nm). A total number of 10,000 cells was analyzed for each sample. The viability of cells after treatment with doxorubicin alone was compared with the viability after treatment with doxorubicin in combination with the test compounds or verapamil (positive control).

### 2.8. Statistical Analysis

All statistical analyses were performed with Statistica version 13 (StatSoft software). The data for doxorubicin accumulation were based at least on four independent experiments performed in duplicate. Cytotoxicity (PI-exclusion assay) data were evaluated from at least three independent experiments performed in duplicate. Data are expressed as the means ± SD. Results were analyzed using the Student’s *t*-test, significance differences: *p* < 0.1; *p* < 0.05; *p* < 0.005.

## 3. Results

### 3.1. Modeling of the Human P-gp

When this project was started, no 3D structure of the human P-gp was available, and therefore, we modeled its structure using the molecular threading server I-TASSER [35,36]. Only recently, the first structure of the human P-gp determined with electron microscopy (EM) has become available (PDB ID: 6C0V) [11]. This was the first human P-gp structure ever disclosed, and it displayed the outward-facing conformation, which was an important contribution with mechanistic implications. More recently, other human P-gp structures obtained by cryo-EM have been published (PDB IDs: 6QEE and 6QEX) [13]. These structures either contain mutations or are bound with ligands or antibodies that could affect the arrangement of the transmembrane domains. They all lack several parts of the sequence due to their high flexibility and have a poor resolution (3.40 Å at best). For all these reasons, the human P-gp cryo-EM structures were not considered here for predicting the binding of inhibitors, although, for comparison, they were tested in the first screening of lignans (see below).

**Figure 1:**
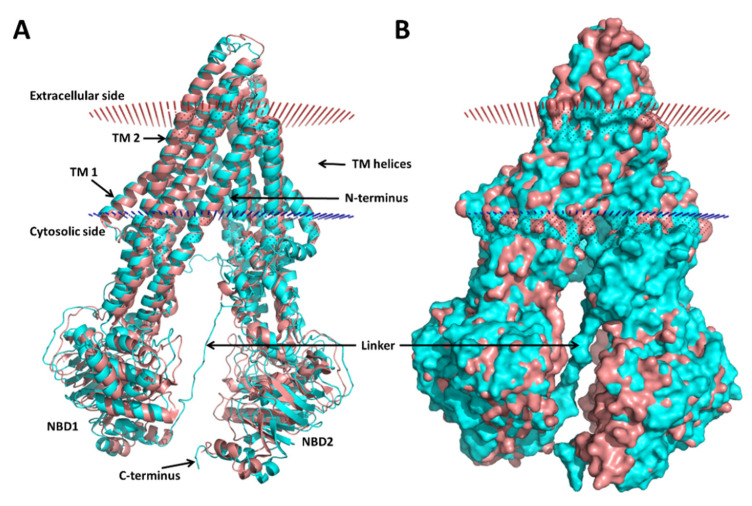
Homology model 1 of the human P-glycoprotein (P-gp) superimposed with a crystal structure of the mouse P-gp (PDB ID: 4M1M). (**A**) Cartoon representation and (**B**) surface representation. The mouse P-gp is colored in salmon, and the model is colored in cyan; the cell membrane boundaries predicted by PPM 2.0 for the model are shown: the red dots represent the extracellular side of the membrane and the blue dots the cytoplasmic side. Several regions of the P-gp are indicated: the nucleotide-binding domains (NBDs) where the ATP binds, the transmembrane (TM) α-helices 1 and 2 (TM 1 and 2); the linker between the two halves (ca. residues 631–684 in the human P-gp numeration), which is missing in the crystallographic structures.

I-TASSER predicted five models based on multiple crystal structures of transporter proteins, the closest one being the mouse P-gp variant with 87% of sequence identity (Supplementary Tables S1 and S2). Besides the I-TASSER default and preferred model (model 1; Figure 1), another model (model 4) also showed favorable and comparable quality scores, as provided by I-TASSER, SAVES, and Swiss-Model (Supplementary Table S3). However, when comparing these structures with a widely used mouse P-gp structure (PDB ID: 4M1M), model 4 presented a much higher RMSD value (6.18 Å) than model 1 (2.41 Å). For this reason, and to prevent using potentially biologically irrelevant conformations of the human P-gp, we discarded model 4, and model 1 was used in all the subsequent analyses.

**Figure 2:**
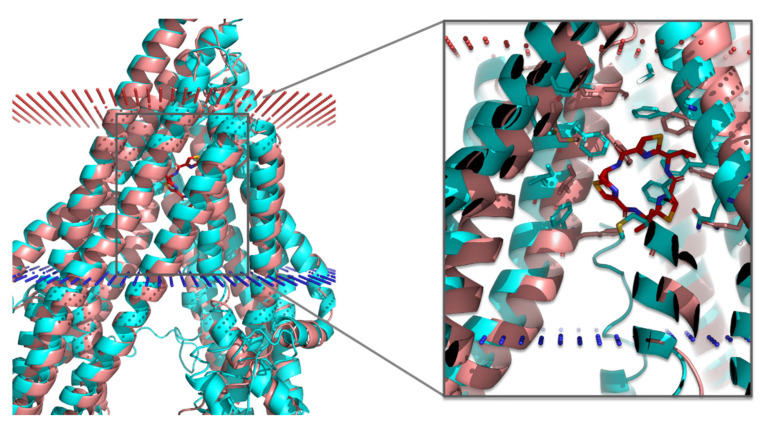
Modulator-binding site (M site) of the P-glycoprotein (P-gp) within the TM region, with superposition of the homology model and the crystal structure of the mouse P-gp bound with the QZ59-*RRR* inhibitor (PDB ID: 3G60). The inhibitor is represented as red sticks, the mouse P-gp is shown in salmon color, and the model in cyan; the cell membrane is represented by the blue (cytosolic side) and red (extracellular side) dots; the numeration is reported according to the human P-gp.

The selected theoretical model is slightly more open in the cytoplasmic region than the mouse variant (Figure 1). The linker region connecting the two symmetric halves of P-gp, which is missing in all crystallographic structures available (residues 627–683 in PDB IDs 4M1M, 3G60, and 3G61), is predicted in our models as a loop (Figure 1). This is in good agreement with previous works [26,70]. The mouse structures also lack the N-terminus (residues 1–30), and all the I-TASSER models (except for model 2, with low-quality scores) displayed the end of this region (residues 1–7) slightly inserted in the transmembrane region, near the binding site (Figure 2). To date, there is no evidence of the correct location of the N-terminus, and this can be a potential source of errors that needs to be handled with caution. However, if proven correct, it may be relevant for an accurate prediction of the ligand binding to P-gp.

One of the binding sites identified in the mouse P-gp (e.g., M site, from PDB ID: 3G60) is quite similar in the homology model, except for the orientation of some of the side chains (Figure 2 and Supplementary Figure S1). The most significant shifts in the side chain positioning or orientation were found for F303, Y307, F343, V982, F983, and M986 (human P-gp numeration, corresponding to F299, Y303, F339, V978, F979, and M982 in the mouse P-gp numeration, respectively; Supplementary Figure S1).

### 3.2. Molecular Dynamics

The structural prediction methods are known to have a relatively high degree of inaccuracy, which can be particularly critical in the case of large biomolecules such as the P-gp (1280 residues). To improve the quality of the 3D model, we carried out an MD simulation in explicit solvent for 500 ns. This MD also served the purpose of testing the stability of our initial structure and diversifying the available conformations of P-gp, and possibly finding more relevant biologically active conformations. To simplify the MD simulation and alleviate the burden of very computationally demanding calculations on an already large system (ca. 207,000 atoms after the solvent was added), we omitted the membrane phospholipid bilayer. Instead of the explicit lipid bilayer, we imposed very mild constraints on the transmembrane helices to prevent unrealistic drifting of the transmembrane domain. These constraints were applied to the backbone of the helices in the transmembrane region, in the form of positional restraints with a force constant of 0.5 kcal mol^−1^⋅Å^−2^ (2.1 kJ⋅mol^−1^⋅Å^−2^). With such low values, these restraints would not prevent low to medium range movements of those helices during MD, should they be necessary for a good equilibration and dynamics of the system. However, they might be sufficient to avoid long-range movements, similarly to what could be expected in the presence of the explicit phospholipid molecules. After an initial conformational readjustment, the system remained stable after ca. 25 ns of simulation. The total RMSD for the backbone atoms remained below 7 Å, oscillating around an average RMSD of 6.3 ± 0.2 Å during the last 400 ns (Supplementary Figure S2). The atomic fluctuations (measured in terms of B-factors; Supplementary Figures S3 and S4) were highest in the linker region (ca. residues 630–690), on a loop region near the N-terminus (ca. residues 17–30) and on one of the extracellular loops (ca. residues 90–100), and medium-high in some of the intracellular loops and short helices between the NBD and TM domains (e.g., ca. residues 500–550).

The major changes observed during the 500 ns MD consisted mainly of a slight closure of the intracellular NDB domains together, compared to the initial model, and a readjustment of the linker chain. Regarding the latter, the linker loop became more folded in itself, becoming more helical, and it showed a downward shift towards the NDB2 domain. This observation is in agreement with a previous study reporting a similar behavior [26].

One of the primary purposes of this MD simulation was to find some P-gp conformation closer to the bioactive form when the protein is bound to inhibitors or modulators, so that it can be used for predictive purposes in virtual screening endeavors. For that, the MD trajectory was clustered according to the orientation of the transmembrane residues, since this is the region where we expect the inhibitors to bind. Therefore, we used the RMSD of those residues as a metric for clustering the trajectories and extract the dominant conformations of P-gp sampled during the MD. We initially constructed five clusters from the MD, but none of them provided reasonable agreements between the respective docking scores and the biological activities for our testing set of lignan compounds (see below). Then, we increased the number to 10 clusters and extracted the respective centroid structures, to which we docked the lignans. Finally, we obtained some satisfactory results. We termed these structures as *cluster 0*–*cluster 9* (the lower the numbers corresponding to the higher populations; Supplementary Figures S5 and S6). We then assessed the structural quality of these cluster structures with the Swiss-Model and SAVES servers (Supplementary Table S4). As expected, we confirmed a significant improvement of all the quality parameters in those structures in comparison to the initial model.

### 3.3. Molecular Docking of the Training Set

**Figure 3:**
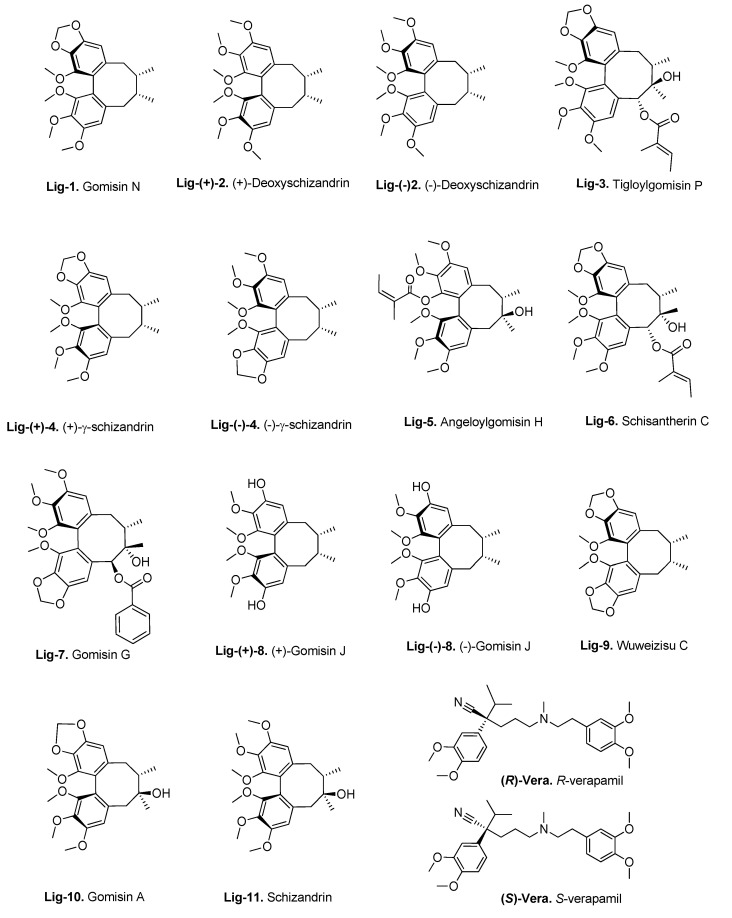
Structures and semitrivial names of the lignans (**lig-1**–**lig-11**) used in the training set and the reference inhibitor verapamil (**(*R*)/(*S*)-vera**) [24].

A set of lignans from a previous study [24] (compounds **lig-1**–**lig-11**; (Figure 3) was chosen to test the suitability of our P-gp structures for predicting the affinity of P-gp inhibitors using molecular docking. Those compounds were docked into the different structures of the human P-gp obtained as previously described: the initial model from I-TASSER (model 1), the structures after the equilibration MD, and the final clusters from MD (structures available in Supplementary material). We also tested two mouse P-gp crystallographic structures and the three human P-gp cryo-EM structures available for comparison. The docking binding modes obtained from AutoDock Vina were re-scored by the empirical scoring function SMINA [64] and by two scoring functions developed based on machine learning techniques, the NNScore [65] and RF-Score-VS [66]. The results were analyzed in terms of the binding energy (ΔG_bind_) or the dissociation constants (K_d_) predicted for the top-ranked conformations as a measure of the compounds’ affinities. For both parameters, the lower they are, the stronger the binding affinity is.

The affinity scores predicted by each tool for the different P-gp structures were correlated with the experimental biological activity, quantified by the percentage of accumulation of an anti-cancer drug, doxorubicin, in HL60/MDR cells, induced by the compounds [24] (Supplementary Table S5). In general, neither AutoDock Vina, SMINA, nor RF-Score-VS provided significant correlations between the docking scores and the percentage of accumulation. The only exception was for the mouse P-gp (PDB ID: 3G60) with RF-Score-VS, which showed quite high Spearman correlation coefficient (*R_S_* = −0.56) but low Pearson correlation (*R_P_* = −0.14). A possible reason for the general lack of correlation is that the compounds do not differ remarkably in their affinities and biological activities, and those docking scores are not sensitive enough to differentiate between them. On the other hand, the NNScore was able to reproduce the affinity trends with relatively good accuracy towards several structures.

Overall, the best correlations were found for the NNScore predictions with *cluster 5* (*R_P_* = −0.5448 and *R_S_* = −0.4505; Table 1 and Supplementary Table S5). These reasonably high correlations suggest that this structure is possibly the best one for predicting the correct binding and the affinity ranking of the inhibitors in our training set of lignans. For this reason, in the subsequent virtual screening, we performed molecular docking on *cluster 5* and a re-scoring with NNScore. Interestingly, the docking calculations on the mouse P-gp structures (PDB ID: 4M1M) re-scored by NNScore also presented reasonable correlations with the biological activities (*R_P_* = −0.3695 and *R_S_* = −0.2044), although not as good as for *cluster 5*. Similar results were obtained with the recent cryo-EM structure of the human P-gp (PDB ID: 6QEE; *R_P_* = −0.3951 and *R_S_* = −0.2659).

The molecular docking predicted, in general, quite strong affinities of the lignans to P-gp, with ΔG_bind_ values ranging between −7.2 and −8.9 kcal/mol (Autodock Vina), and *K*_d_ values in the sub-micromolar range (Table 1). When we compare the predicted *K*_d_ affinities and the experimental biological activities, the trends are not perfectly matched and show a few obvious disagreements, which is the reason for the lack of perfect correlations, as described above. NNScore predicted the highest affinity for **lig-(+)-4** (*K*_d_ of 17.5 nM), while the best one (**lig-1,** *K*_d_ of 28.3 nM) ranked third. However, it correctly identified the least active lignans (**lig-10** and **lig-11**, with *K*_d_ of 257.0 and 359.8 nM, respectively). Such irregularities are not unexpected since the docking programs are rather designed for the selection enrichment than for the precise differentiation between similar biological activities (the 2.3-fold difference between the best and worst inhibitors is not very high). Regarding the reference inhibitor verapamil, it is composed of a racemic mixture of two enantiomers, the *R*- and *S*-verapamil (Figure 3). As expected, these enantiomers seem to bind P-gp differently (in terms of binding conformations and affinities), but both were predicted to have *K*_d_ values one order of magnitude lower than the best lignans (Table 1). Such difference may be due to the very different types of chemical scaffolds present in verapamil and the lignans, which lead to a shift in the *K*_d_ scales for these two classes of compounds. The same situation might apply when comparing any other classes of molecules.

**Figure 4:**
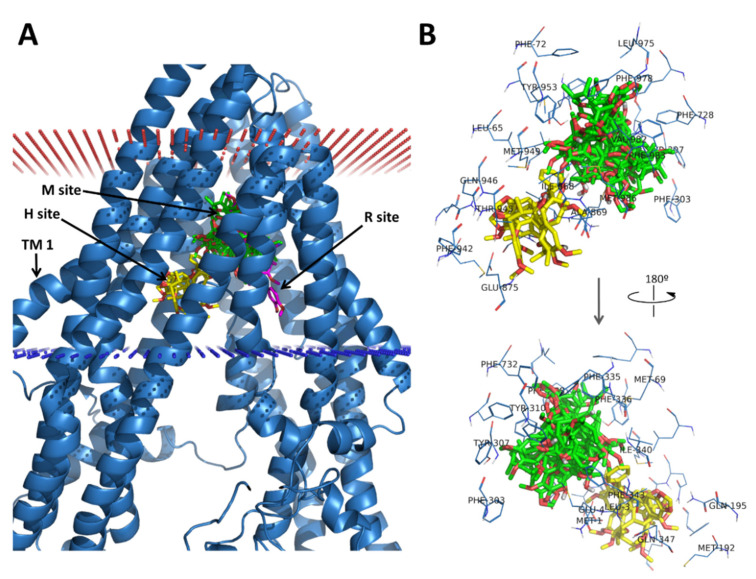
Docking of the lignans and verapamil into the human P-glycoprotein (P-gp). (**A**) The different binding sites within the TM region; (**B**) detail of the interacting residues at the M and H sites. *R*- and *S*-verapamil (magenta sticks) bound at the M/R site, lignans **lig-1**, **lig-7**, and **lig-10** (yellow sticks) bound preferentially at the H site, and the remaining lignans (green sticks) bound at the M site. The P-gp structure used for docking (cluster 5 from MD) is represented by the blue cartoon or blue lines, while the cell membrane is represented by the blue (cytosolic side) and red (extracellular side) dots.

The preferred binding positions of the docked lignans, as predicted by the NNScore, were mostly located at the M site, as the QZ59-*RRR* inhibitor bound to the mouse P-gp (PDB ID: 3G60; Figure 4). However, some of them bound preferentially at the H site (**lig-1**, **lig-7**, **lig-10**). On the other hand, verapamil was bound between the M and the R site, which is in agreement with previous results [9]. The interactions formed by these inhibitors at the M site have a strong aromatic or hydrophobic character, provided mainly by the multiple phenylalanine, tyrosine, leucine, and methionine residues in the binding site (namely Leu65, Met69, Y310, F335, F336, I340, F343, F978, and F983, and Y953), as shown in Figure 4. In some cases, the hydroxyl groups of Y310 and Y953 could form hydrogen bonds with the ether- or hydroxyl-*O* atoms of the inhibitors. On the other hand, the hydroxyl groups present in the lignans, such as the 7-hydroxyl (**lig-3**, **lig-5**, **lig-6**, **lig-7**, **lig-10,** and **lig-11**,) or the 3,12-dihydroxy (in **lig-(+/-)-8**), did not contribute much to improve the binding affinity, as they were rarely involved in favorable interactions with the protein. The only exceptions were **lig-3** and **lig-6**, whose OH groups formed hydrogen bonds with the phenolic OH group of Y953 and Y310, respectively. Globally, these findings are in good agreement with the QSAR model previously described for these lignans [24].

### 3.4. Virtual Screening of Natural Flavonoids

To validate our previously selected model and find new potentially active P-gp inhibitors, we performed a structure-based virtual screening with a set of 87 flavonoids found in the literature (compounds **1**–**87**; Supplementary Figure S7) [21,67,71,72,73,74,75]. As mentioned above, *cluster 5* from the MD simulation was used as the receptor for docking these compounds. The docking results from AutoDock Vina were re-scored by NNScore, previously found to be the most reliable affinity predictor (*K*_d_ values) for the initial set of lignans.

**Figure 5:**
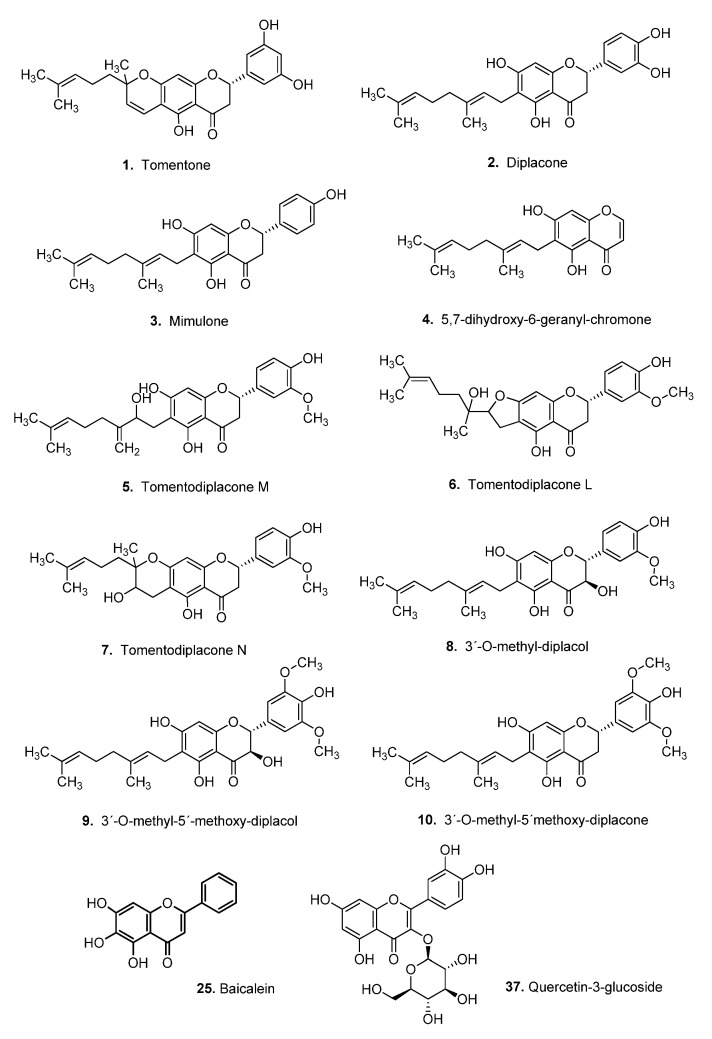
Structures and semitrivial names of the flavonoids experimentally screened in this work (**1**–**10**) and the two top-ranked in the virtual screening (**25** and **37**).

The results showed that many of the tested flavonoids were predicted to have very high affinity towards P-gp, with *K*_d_ values in the low nanomolar concentration ranges (*K*_d_ ≥ 1.16 nM; Table 2 and Supplementary Table S6). Among these, the compounds with both highest *K*_d_ and ΔG scores (i.e., lower values) can be especially interesting for further experimental testing, when available, namely **25** and **37**. Concerning compound **25**, baicalein, this result was consistent with several previous studies that showed its P-gp-inhibitory effect on P-gp overexpressing cells [75,76]. Furthermore, our results on the potential of **37** (quercetin-3-glucoside) to interact with P-gp are new and suggest the possibility to test this compound on some cellular systems. Several screened flavonoids (namely **3**, **10**, **25**, and **37**) seemed to be potentially stronger binders than any of the lignans in the training set (*K*_d_ ≤ 17.5 nM). Moreover, all of the 10 flavonoids to be experimentally tested (compounds **1**–**10**; Figure 5) were predicted to be better binders than the worst lignan screened (*K*_d_ ≤ 359.8 nM). We also find that these ten compounds are among the 50% best-ranked flavonoids, with the worst prediction for compound **6** with *K*_d_ 36.6 nM. Among these, the top-ranked five can be ordered as: **10** > **3** > **4** > **7** > **5**. We also analyzed the best binding scores of the different flavonoids towards any structure in our P-gp ensemble (i.e., not just *cluster 5*).

To investigate the binding modes of the flavonoids to the P-gp, we analyzed more in detail the docked conformations of the experimentally tested (**1–10**) and the two best-ranked compounds (**25** and **37**). All these flavonoids were predicted to bind either at the M site, as most of the lignans described above, or in between the M and the R site (**5**, **6**, **8**, **9**, **10**), as observed for verapamil (Supplementary Figure S8). The dominant interactions were also the aromatic and hydrophobic contacts with the several phenylalanine, tyrosine, leucine, and methionine residues. In this case, several hydrogen bonds were formed between the hydroxyl groups of the inhibitors and the side chains of Y307, Q725, and Y953, or the backbone of F336 and I340 at the M site. The R site is richer in polar residues, and the binding of the inhibitors here can form a larger number of hydrogen bonds, namely with E4, D6, N8, Q990, and Q838 (Supplementary Figure S8). Such is also the case of compound **10** (Supplementary Figure S9A). The best-ranked flavonoids (**25** and **37**) were bound only at the M site, forming several favorable hydrophobic contacts and hydrogen bonds with the protein (Supplementary Figure S9B).

### 3.5. Biological Activity of Flavonoids

**Figure 6:**
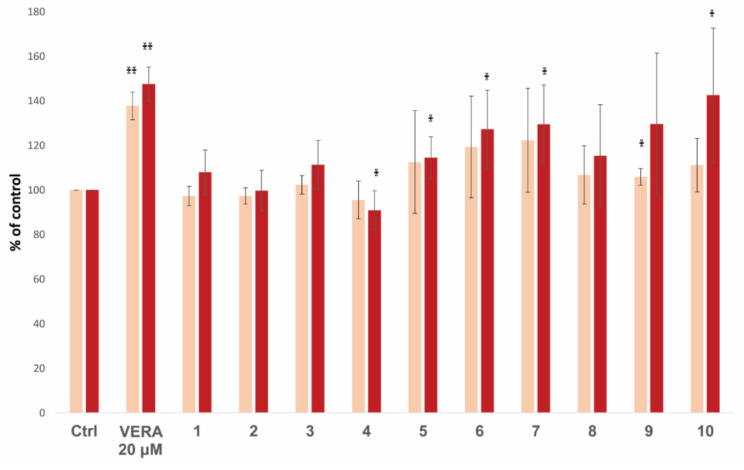
Effect of flavonoids (**1**–**10**) at a concentration of 10 μM (pink) and 20 μM (red) on the intracellular accumulation of doxorubicin in resistant HL60/MDR cells. The accumulation was determined after 1 h of incubation of cells with doxorubicin (10 μM) in the presence or absence of the flavonoids **1**–**10** or verapamil (VERA, 20 μM used as a positive control) using flow cytometry doxorubicin accumulation assay. Results were expressed as the percentage ratio of the doxorubicin fluorescence in the presence of each modulator to the fluorescence of doxorubicin alone (Ctrl). The data show means ± SD of at least four independent experiments (* *p* < 0.05; ** *p* < 0.005).

The ability of flavonoids to inhibit P-gp was determined by measuring the fluorescent substrate doxorubicin accumulation within the resistant HL60/MDR cells using flow cytometry. The results are shown in Table 2 and Figure 6.

The available flavonoids, compounds **1–10**, were evaluated and compared to the reference P-gp inhibitor verapamil. A statistically significant increase of the intracellular doxorubicin was determined in the presence of both verapamil (20 µM) and flavonoids **5**, **6**, **7** and **10** at a concentration of 20 µM, and flavonoid **9** at the concentration of 10 µM. (Table 2 and Figure 6). The remaining flavonoids did not show a significant effect on the doxorubicin accumulation.

**Figure 7:**
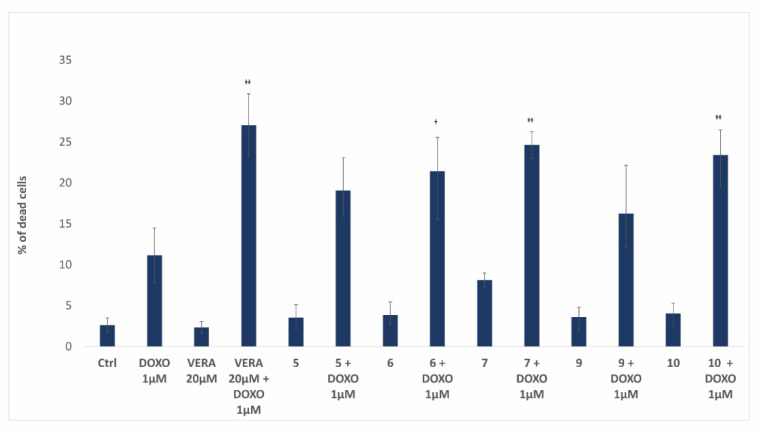
Viability of HL60/MDR cells treated for 48 h by doxorubicin (DOXO) at a concentration of 1 μM in the presence or absence of the flavonoids (**10**, **9**, **7**, **6** and **5**) or verapamil (VERA), all at a concentration of 20 μM. The viability of cells after treatment with doxorubicin alone was compared with viability after treatment with doxorubicin in combination with the flavonoid or verapamil used as a positive control. The data show the means ± SD of at least three independent experiments. (* *p* < 0.05; ** *p* < 0.005).

We used a PI exclusion assay to reveal the effect of flavonoids on doxorubicin-mediated cytotoxicity. We treated resistant HL60/MDR cells with doxorubicin in sub-toxic concentration (1 µM) together with the best flavonoids (**5**, **6**, **7**, **9,** and **10**) from the accumulation assay at a concentration of 20 µM for 48 h. Upon the doxorubicin treatment, only 11% of the cells were dead. The flavonoids alone were non-toxic (2.3–4% dead cells), but they potentiated the toxicity of doxorubicin (16.3–24.6% of dead cells upon combined treatment). The supporting effect of verapamil (used as a positive control) and flavonoids **6**, **7**, and **10** on doxorubicin toxicity were statistically significant (Figure 7). 

Overall, these results show that the tested flavonoids potentiate the antiproliferative effect of doxorubicin on resistant cancer cells by increasing the amount of the drug inside the cells.

## 4. Discussion

Cancer is the second most common cause of death, with 18.1 million new cases and about 9.6 million deaths in 2018 [77]. Chemotherapy is the mainstay of most cancer treatments. However, a significant number of patients develop resistance to a broad spectrum of structurally different anti-cancer drugs, with so-called multidrug resistance (MDR). Overexpression of ABC-transporters, which mediate the efflux of anti-cancer drugs out of the cells, is one of the main reasons for MDR [1,78]. P-gp is the ABC-transporter that is most often responsible for MDR. The administration of inhibitors of the membrane pumps together with anti-cancer drugs is the major strategy to overcome MDR. Therefore, the search for inhibitors of this protein is an important goal to increase the efficiency of anticancer therapy. However, the inhibitors known so far have serious side effects. Hence, seeking new inhibitors of P-gp without such drawbacks is of utmost importance. In this respect, the development of in silico models for the identification of P-gp inhibitors is of great interest in the field of cancer drug discovery.

Aiming at creating a new predictive methodology for the virtual screening of potential P-gp inhibitors based on the correlation of docking scores with the anti-MDR biological properties, we started by modeling the structure of the human P-gp. Due to the lack of a crystal structure of the human P-gp, we used molecular threading with I-TASSER. This allowed us to have a structural estimation of the missing segments in the majority of the studies performed to date, namely the terminal sections and the linker between the two pseudo-symmetrical units, which may have important functional roles and influence the dynamical behavior of the P-gp [70,79]. From the different models obtained, we selected the most reliable one according to several quality assessment parameters. This model, showing the standard inward-facing conformation similar to most known P-gp homologs (Figure 1), was refined by MD simulation, which improved its structural quality. The MD was then clustered for obtaining a representative dynamical ensemble of P-gp. The MD also revealed the most flexible parts in the human P-gp, which was in agreement with the general assumption that the linker is highly flexible and disordered [10,70].

Several studies have demonstrated that dibenzocyclooctadiene lignans, natural products from *S. chinensis*, can reverse P-gp- or MRP1-mediated multidrug resistance [80,81,82]. In a previous study, we reported a new set of dibenzocyclooctadiene lignans active against MDR in leukemia HL60/MDR cells overexpressing P-gp [24]. We also identified the structural characteristics of dibenzocyclooctadiene lignans essential for the P-gp inhibition. In the present study, we performed molecular docking of these lignans (**lig-1**–**lig-11**; see Figure 3) against the representative P-gp structures obtained from the MD ensemble. The aim was to find the best P-gp conformation in that ensemble for predicting the correct binding of the lignan inhibitors and the best scoring function to predict their affinity order. This was achieved by assessing the correlations between the calculated binding affinities and the respective biological activities (measured by the intracellular accumulation of doxorubicin in resistant HL60/MDR cells). The system showing the best correlations (the MD *cluster 5* as the receptor and NNscore as the re-scoring function) was then selected for all the further analyses. The screened lignans were predicted to bind preferentially at the M site of the drug-binding site, as other known inhibitors (e.g., the QZ59-*RRR* inhibitor found in PDB ID 3G60), or at the H site. Verapamil seemed able to bind at both M and R sites, in agreement with previous findings [9]. Our preliminary quantitative structure−activity relationship (QSAR) analysis of the lignans [24] revealed three main structural features contributing to a more potent P-gp inhibition: the presence of a 1,2,3-trimethoxy moiety, a 6-acyloxy group, and the absence of a 7-hydroxy group. According to the docking results, the preferred binding site of the lignans (M site) has a predominantly hydrophobic nature, with multiple aromatic (Phe and Tyr) and hydrophobic (Ile and Leu) residues. This is ideal for accommodating the hydrophobic methoxy and acyloxy groups present in the lignans. Moreover, we found a general lack of favorable interactions between the 7-hydroxyl or the 3,12-dihydroxy groups with the binding site residues. Altogether, these results are in good agreement with our previous QSAR model.

Taking advantage of the virtual screening methodology developed for the lignans, we decided to use it for screening a different library of natural compounds, composed of 87 flavonoids found in the literature (Supplementary Figure S7). Thus, we obtained a ranking of those flavonoids according to their predicted affinities towards the human P-gp, which should correspond to their ability to inhibit the transport of drugs by this protein. Many of the flavonoids screened were predicted more potent binders of P-gp than the tested lignans (with *K*_d_ values in the low nanomolar range). We had access to 10 geranylated flavonoids obtained from the fruit of *P. tomentosa* (flavonoids **1**–**10**; see Figure 5) [21,67]. They represent a part of the lipophilic resinous exudate, located on the fruit surface, and can be successfully obtained by a relatively simple separation procedure. We have previously demonstrated the toxicity of these flavonoids towards a panel of cancer cell lines [68,83]. Here, we studied the effect of these available flavonoids on multidrug-resistant cancer cells. Of all the tested flavonoids, compound **10** revealed the best performance in the accumulation assays (ability to increase the doxorubicin accumulation in the cells). It reached activity levels close to the reference inhibitor verapamil (Table 2). It was also the best flavonoid in increasing the cytotoxicity of doxorubicin. In the virtual screening, compound **10** was the best among the flavonoids assayed, indicating a perfect agreement with the biological results. Flavonoid **7** was the second-best in inducing doxorubicin accumulation in the cells and potentiate its cytotoxic effects, and it ranked fourth in the virtual screening (among the tested compounds; Table 2).

A second interesting result from the virtual screening is the top-ranked compounds, namely **25** (baicalein) and **37** (quercetin-3-glucoside). Several authors have described the inhibitory effect of baicalein on P-gp [75,76], thus confirming such prediction from our virtual screening. However, to our knowledge, the result suggesting that quercetin-3-glucoside can interact with P-gp is new. Its effect on P-gp was studied only by Kitagawa et al. [84], who showed that quercetin-3-glucoside, in contrast to baicalein, did not increase the accumulation of Rhodamine-123 in P-gp overexpressing KB-C2 cells. On the other hand, it has been demonstrated that quercetin-3-glucoside can elicit antiproliferative effect in human breast cancer cells [85]. Therefore, it would be important to further test quercetin-3-glucoside (**37**) in different types of P-gp overexpressing cells, in order to clarify whether this compound could present biological activity in those cellular systems. We propose that other highly ranked flavonoids from our virtual screening may also show great potential as P-gp inhibitors and should be experimentally tested when available.

Surprisingly, some of the flavonoids predicted to have high affinity with P-gp were revealed to be inefficient as MDR modulators in the accumulation assays. Namely, compound **3**, which was predicted to be a strong binder, after **10**, and compounds **1**, **4,** and **5**, which were predicted to have high affinities towards P-gp (Kd ≤ 16.8 nM) in the virtual screening, revealed only slight or no biological effects. The possible reasons for the failure in predicting these biological activities are manifold: (1) the P-gp structure with best correlations for the lignans may be non-optimal for predicting the binding of flavonoids due to their different chemical scaffolds; (2) the mechanism of P-gp inhibition by the flavonoids is different from that of the lignans; (3) the bioavailability and possible metabolism of flavonoids in the cell is the limiting factor to their activity over their inhibitory activity with P-gp. Flavonoid compounds with lipophilic side chains are relatively insufficiently studied from the cell metabolism point of view and their behavior in cellular systems. Information about possible oxidation, sulfation, and glucuronidation modifications of the prenyl side chains is available for some prenylated flavonoids. However, this was obtained from the analysis of full metabolic profiles from in vivo studies and does not represent how those compounds behave in cells [86]. Similar examples are the metabolic studies done on hop prenylated flavonoids using microbes [87]. However, at this moment, the changes due to cellular metabolism on the tested structures are unpredictable and will need experimental evaluation. To assess precisely which of these factors are the true culprits for the poor estimation of the flavonoid activity would require extensive calculations (such as more extended MD simulations, accurate free energy calculations, or MDs with the P-gp bound to the inhibitors) and further biological experiments to reveal the metabolism of those compounds.

The set of flavonoids screened is still relatively small to obtain a relevant structure-activity relationship. However, a clearly important feature is the flavonoid ring B, as compound **4**, lacking this structural moiety, showed the lowest biological effect. The more extensive substitution of ring B (e.g., hydroxy and methoxy; hydroxy and dimethoxy) also seems more favorable to biological activity. The biologically effective concentrations of the tested flavonoids are much higher (10 to 20 µM) than the concentrations predicted by the virtual screening (*K*_d_ in the nanomolar range). These discrepancies may again be due to the differences in the bioavailability of these flavonoids. The actual intracellular concentration of these compounds is influenced by their binding to the proteins contained in the medium and by their ability to penetrate through the cell membrane. Their concentration may also be reduced by their metabolism by cellular enzymes. Nonetheless, as demonstrated by both in silico and in vitro assays, the potential of flavonoids to decrease the resistance of cancer cells to doxorubicin is promising. However, their bioavailability, behavior in a culture medium, and potential metabolism must be evaluated to understand some discrepancies between the modeling results and the in vitro cellular assays.

## 5. Conclusions

Here, we predicted a model of the full-length human P-gp, assessed its flexibility and structural diversity, and established a methodology for the structure-based virtual screening of P-gp inhibitors. We disclosed the binding modes of a set of lignans, which previously demonstrated to be active inhibitors of P-gp, to the human P-gp, and confirmed the importance of several structural features. To find potentially new P-gp inhibitors, we virtually screened a library of other natural products, the flavonoids. We tested 10 of these flavonoids in vitro against resistant cancer cells overexpressing P-gp. At least two of these flavonoids were able to increase both the accumulation and the cytotoxicity of doxorubicin in resistant cancer cells. The results from the in vitro experiments did not confirm precisely the computational predictions due to several possible reasons. Despite some limitations, several successful predictions were attained from our computational approach. Collectively, our results demonstrate the enormous potential of molecular modeling and virtual screening methods, namely in prioritizing candidates and finding new effective drugs to prevent multidrug resistance in the treatment of cancer or other diseases.

## Figures and Tables

**Table 1:** Scoring results for the docking calculations providing the best agreement between the experimental data for the lignan inhibitors and the respective docking ^a^.

	Biological Activity ^b^	AutoDock Vina	SMINA	NNScore	RF-Score-VS
Compound	(% Accumulation)	ΔG_bind_ (kcal/mol)	ΔG_bind_ (kcal/mol)	*K*_d_ (nM)	*K*_d_ (nM)
**Lig-1**	283	−7.6	−7.60	28.3	702.6
**Lig-(-)-2**	277	−7.6	−7.57	71.4	567.8
**Lig-(+)-2**	264	−7.3	−7.30	144.1	631.9
**Lig-3**	234	−7.3	−8.32	48.3	678.0
**Lig-(+)-4**	232	−7.8	−7.81	17.5	626.9
**Lig-(-)-4**	152	−7.8	−7.90	50.7	723.8
**Lig-5**	202	−7.2	−7.41	46.7	737.1
**Lig-6**	201	−8.9	−9.08	67.1	583.8
**Lig-7**	199	−8.0	−9.02	17.9	637.3
**Lig-(-)-8**	191	−7.2	−7.61	283.2	704.3
**Lig-(+)-8**	156	−7.2	−7.55	181.0	814.2
**Lig-9**	187	−8.4	−8.45	41.2	742.7
**Lig-10**	162	−7.3	−7.85	257.0	554.0
**Lig-11**	125	−7.3	−7.40	359.8	609.8
Pearson *R_P_* ^b^		−0.0308	0.0808	−0.5448	−0.2009
Spearman *R_S_* ^c^		−0.0850	0.1033	−0.4505	−0.2088
**(*R*)-vera**	239	−7.9	−8.06	0.74	834.5
**(*S*)-vera**	239	−7.3	−8.15	0.46	845.8

^a^ Obtained for cluster 5 from MD; stronger inhibition corresponds to higher % accumulation, more negative binding energy, ΔG_bind_, and lower dissociation constant, K_d_; ^b^ intracellular doxorubicin accumulation (in %) in HL60/MDR cells after pretreatment with 25 µM of inhibitor; ^b^ Pearson correlation coefficient (R_P_) between the biological activities and the docking scores; ^c^ Spearman’s rank correlation coefficient (R_S_), which describes the relationship between the biological activities and the docking score using a monotonic function, to assess their consistency in terms of the trends; in all cases, more negative *R* values correspond to better correlations.

**Table 2:** Virtual screening affinity predictions (*K*_d_ and ΔG_bind_) for the top-ranked and the experimentally tested flavonoid compounds, and the respective biological activities.

Virtual Screening ^a^				Biological Activity ^b^	
*Ranking*	Compound	*K*_d_ (nM)	ΔG_bind_ (kcal/mol)	% Accumulation (10 µM)	% Accumulation (20 µM)
*1*	**25**	1.16	−7.7	n.d.	n.d.
*2*	**37**	1.27	−9.0	n.d.	n.d.
*…*	…				
*14*	**10**	3.49	−9.5	**111.2 ± 12.0**	**142.5 ± 30.2**
*15*	**3**	3.80	−9.9	102.3 ± 4.2	111.3 ± 11.0
*25*	**4**	9.62	−8.2	95.5 ± 8.5	90.9 ± 8.9
*31*	**7**	14.3	−10.1	**122.3 ± 23.4**	**129.5 ± 17.6**
*32*	**5**	15.3	−9.4	112.5 ± 23.1	**114.5 ± 9.4**
*33*	**1**	16.8	−9.5	97.3 ± 4.3	107.9 ± 10.1
*34*	**2**	17.8	−9.3	97.3 ± 3.7	99.7 ± 9.1
*40*	**9**	26.0	−9.4	105.9 ± 3.7	**129.6 ± 31.9**
*42*	**8**	28.4	−9.4	106.7 ± 13.1	115.3 ± 23.0
*44*	**6**	36.6	−9.9	119.3 ± 22.9	**127.2 ± 17.6**
*Best lignan ^c^*	**Lig-(+)-4**	17.5	−7.6	n.d.	283 ± 33 (Lig-1) ^d^
*Worst lignan ^c^*	**Lig-11**	359.8	−7.3	n.d.	125 ± 17 (Lig-11) ^d^
	***(R)*-vera** ***(S)*-vera**	0.74 0.46	−7.9 −7.3	137.7 ± 6.2	147.5 ± 7.7 239 ± 30 ^d^

^a^ The compounds are ranked by the predicted *K*_d_ affinity scores (from NNscore) with *cluster 5*, while the respective ΔG_bind_ (from AutoDock Vina) is presented for comparison; ^b^ intracellular doxorubicin accumulation (in %) in HL60/MDR cells is the mean value ± SD from at least four independent experiments performed in duplicate, after pretreatment with the flavonoids at a concentration of 10 µM and 20 µM; ^c^ for comparison, the best and worst results obtained in the training set of lignans and verapamil are presented (compound specified in brackets); ^d^ from ref. [24] with 25 µM of inhibitor instead of 20 µM. The significant biological activities are highlighted in bold; n.d. means “not determined”.

## Data Availability

Not applicable.

## Supplementary Materials

The following are available online at https://www.mdpi.com/article/10.3390/biomedicines9040357/s1: Supplementary Figures S1 to S9, Supplementary Tables S1 to S6, and all the P-gp models used in this study (I-TASSER model 1, I-TASSER model 4, model 1 with the membrane description (PPM), Equil.MD, and *cluster 0–cluster 9* from the MD).
