# Supplementary material for: Screening of Natural Compounds as P-Glycoprotein Inhibitors against Multidrug Resistance
Source: Biomedicines. 2021 Mar 30;9(4):357. doi: 10.3390/biomedicines9040357 (PMC8066904; doi:10.3390/biomedicines9040357)
Supplement: Supplementary file 1 [file biomedicines-09-00357-s001.zip › biomedicines-1157903-supplementary_r1.pdf]

Screening of Natural Compounds as P-Glycoprotein Inhibitors Against Multidrug Resistance

Sérgio M. Marques[1,2], Lucie Šupolíková[3] , Lenka Molčanová[4], Karel Šmejkal[4], David Bednar[1,2,*] and Iva Slaninová[3,*]

[1] Loschmidt Laboratories, Department of Experimental Biology and Research Centre for Toxic Compounds in the Environment RECETOX, Faculty of Science, Masaryk University, Kamenice 5/A13, 625 00 Brno, Czech Republic; smar96@gmail.com (S.M.M); davidbednar1208@gmail.com (D.B.)

[2] International Clinical Research Center, St. Anne's University Hospital Brno, Pekarska 53, 656 91 Brno, Czech Republic; smar96@gmail.com (S.M.M); davidbednar1208@gmail.com (D.B.)

[3] Department of Biology, Faculty of Medicine, Masaryk University, Kamenice 5/A6, 625 00 Brno, Czech Republic; supolikova@med.muni.cz (L.Š.); ipokorna@med.muni.cz (I.S.)

[4] Department of Natural Drugs, Faculty of Pharmacy, Masaryk University, Palackého 1946/1, Brno, Czech Republic; karel.mejkal@post.cz (K.Š.); lenka.molcanova1993@gmail.com (L.M.)

* Correspondence: ipokorna@med.muni.cz (I.S.); davidbednar1208@gmail.com (D.B.); Tel.: +420-549496985 (I.S.); +420-549492616 (D.B.)

Supplementary Material

Index

1. Homology modelling 2
2. Molecular dynamics 5
   2.1 Stability and flexibility 5
   2.2 Clustering 7
3. Molecular docking with the training set 10
   3.1 Evaluation of the docking method 10
4. Virtual screening 11
   4.1 Structures of the flavonoid compounds 11
   4.2 Virtual screening results with the flavonoids 16
   4.3 Binding mode of the flavonoids 19

1. Homology modelling

Table S1. Top 10 threading templates used by I-TASSER to construct the models of the human P-gp.

Rank[b]	PDB Hit	Iden1[c]	Iden2[d]	Cov[e]	Norm. Z-score[f]
1	3G61-A	0.88	0.82	0.92	6.74
2	4M1M-A	0.88	0.82	0.93	13.33
3	4F4C-A	0.45	0.45	0.96	7.68
4	5TWV	0.18	0.21	0.88	2.08
5	5TWV	0.18	0.21	0.88	1.51
6	3G61-A	0.89	0.82	0.92	10.84
7	5TWV	0.19	0.21	0.78	2.46
8	3G61-A	0.88	0.82	0.92	6.82
9	3G5U-A	0.87	0.69	0.78	7.69
10	3G61-A	0.89	0.82	0.92	39.79

[b]Rank of templates represents the top ten threading templates used by I-TASSER; [c]Ident1 is the percentage sequence identity of the templates in the threading aligned region with the query sequence; [d]Ident2 is the percentage sequence identity of the whole template chains with query sequence; [e]Cov represents the coverage of the threading alignment and is equal to the number of aligned residues divided by the length of query protein; [f]Norm. Z-score is the normalized Z-score of the threading alignments. Alignment with a Normalized Z-score >1 mean a good alignment and vice versa.

Table S2. Identified structural analogues in PDB.

Rank[a]	PDB Hit	TM-score	RMSD[b]	IDEN[c]	Cov[d]
1	4M1M-A	0.886	2.57	0.874	0.928
2	4F4C-A	0.851	4.24	0.411	0.950
3	5TWV-B	0.682	5.96	0.15	0.833
4	3QF4-B	0.414	4.01	0.293	0.454
5	3WME-A	0.412	3.73	0.366	0.449
6	4MYC-A	0.41	4.32	0.243	0.459
7	4MRN-A	0.397	4.58	0.243	0.448
8	2YL4-A	0.394	4.11	0.342	0.435
9	3B5X-A	0.369	5.16	0.264	0.428

[a]The ranking of proteins is based on the TM-score of the structural alignment between the query structure and known structures in the PDB library; [b]the root-mean-square deviation (RMSD) between residues that are structurally aligned by TM-align; [c]IDEN is the percentage sequence identity in the structurally aligned region; [d]Cov represents the coverage of the alignment by TM-align and is equal to the number of structurally aligned residues divided by length of the query protein.

Table S3. Quality-assessment of the I-TASSER models as predicted by different validation methods.

	**Model 1**	**Model 2**	**Model 3**	**Model 4**	**Model 5**
C-score[a]	0.20	-0.26	-0.06	0.27	-4.07
TM-score[a]	0.74 ± 0.11	-[b]	-[b]	-[b]	-[b]
Estimated RMSD[a]	9.0 ± 4.6 Å	-[b]	-[b]	-[b]	-[b]
PROVE[c]	ERROR[d]	Pass	Pass	Pass	Pass
ERRAT[c]	92.762	84.343	90.412	97.792	88.022
VERIFY3D[c]	ERROR[e]	ERROR[e]	ERROR[e]	ERROR[e]	ERROR[e]
QMEAN[f]	-5.99	-9.66	-7.63	-4.93	-7.79
MolProbity score[f]	3.05	3.65	3.40	2.63	3.13
Ramachandran favoured[f]	84.05%	76.38%	80.98%	85.74%	81.90%
Ramachandran outliers[f]	6.13%	11.96%	6.90%	4.45%	4.91%
Ramanchandran plots (red dots are outliers)[f]	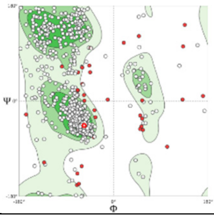	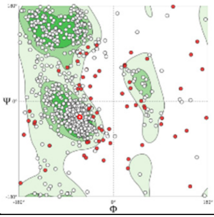	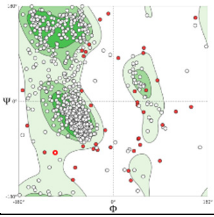	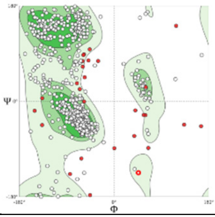	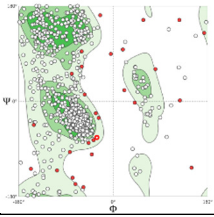
RMSD of C_α from 4M1M[g]	2.41 Å	5.13 Å	0.80 Å	6.18 Å	5.23 Å

[a]Predicted by I-TASSER; [b]not determined; [c]calculated with SAVES; [d]the total number of buried outlier protein atoms was 278 (5.9%) of scored atoms; [e]less than 65% of the amino acids have scored >= 0.2 in the 3D/1D profile; [f]calculated with the Swiss-Model Structure Assessment; [g]RMSD of all C_α atoms from the crystal structure mouse P-gp without bound ligands (PDB ID: 4M1M-chain A) calculated with PyMOL 2.3.2.

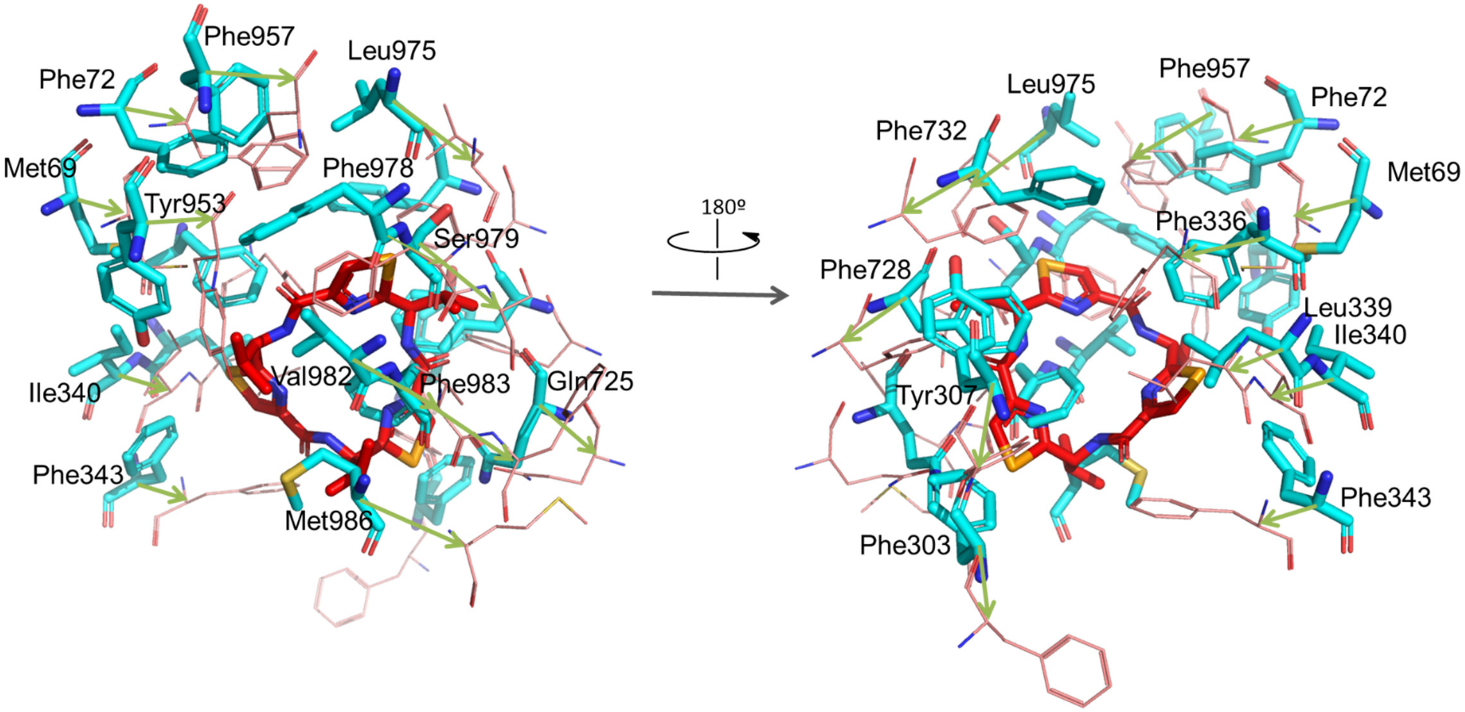

Fig. S1. Modulator-binding site (M site) of the P-gp within the TM region, with superposition of the homology model and the crystal structure of the mouse P-gp bound with the QZ59-*RRR* inhibitor (PDB ID: 3G60). The inhibitor is represented as red sticks, the residues of the P-gp model are shown as cyan sticks and the mouse residues as salmon lines; the numeration is reported according to the human P-gp sequence; the green arrows show the shift of the C_α atoms from the human P-gp to the respective residues in the mouse P-gp homologue.

2. Molecular dynamics

2.1 Stability and flexibility

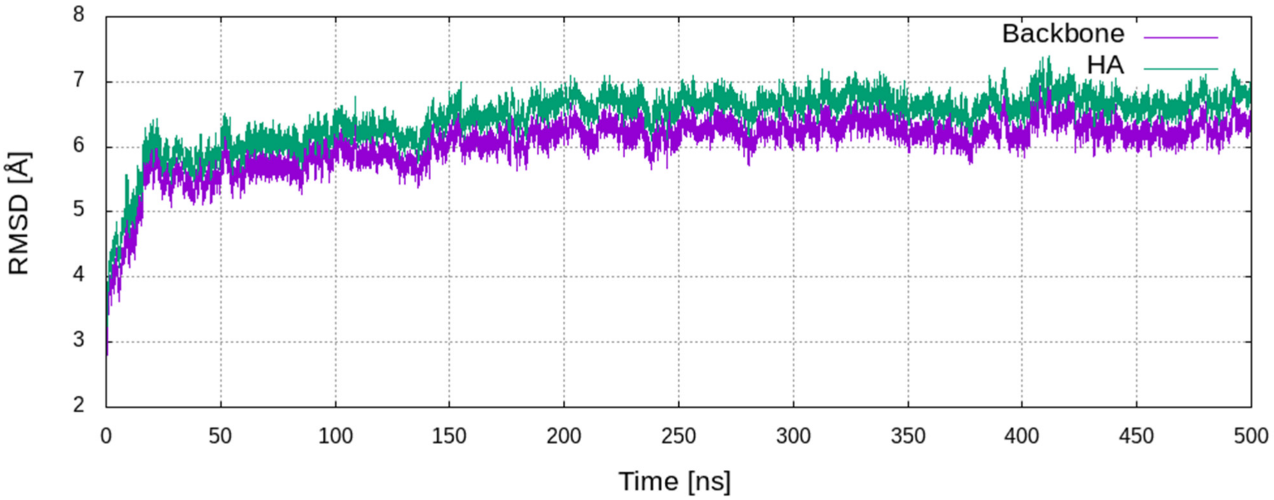

Fig. S2. Evolution of the RMSD of the backbone and heavy atoms (HA), with respect to the initial model 1 structure, during the MD simulation.

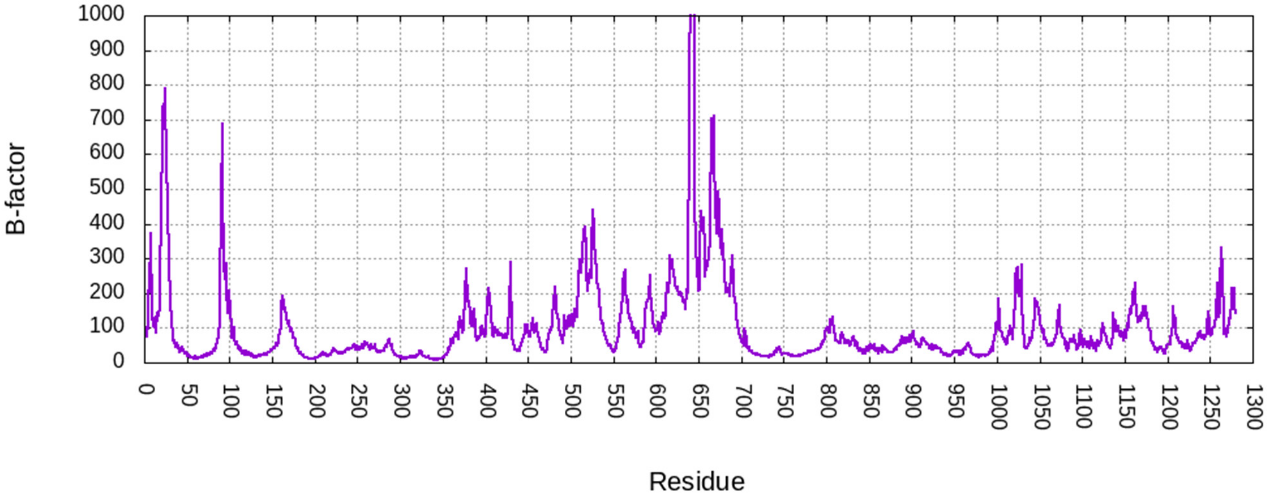

Fig. S3. B-factors by residue of the P-gp during the MD simulation, calculated for the backbone atoms.

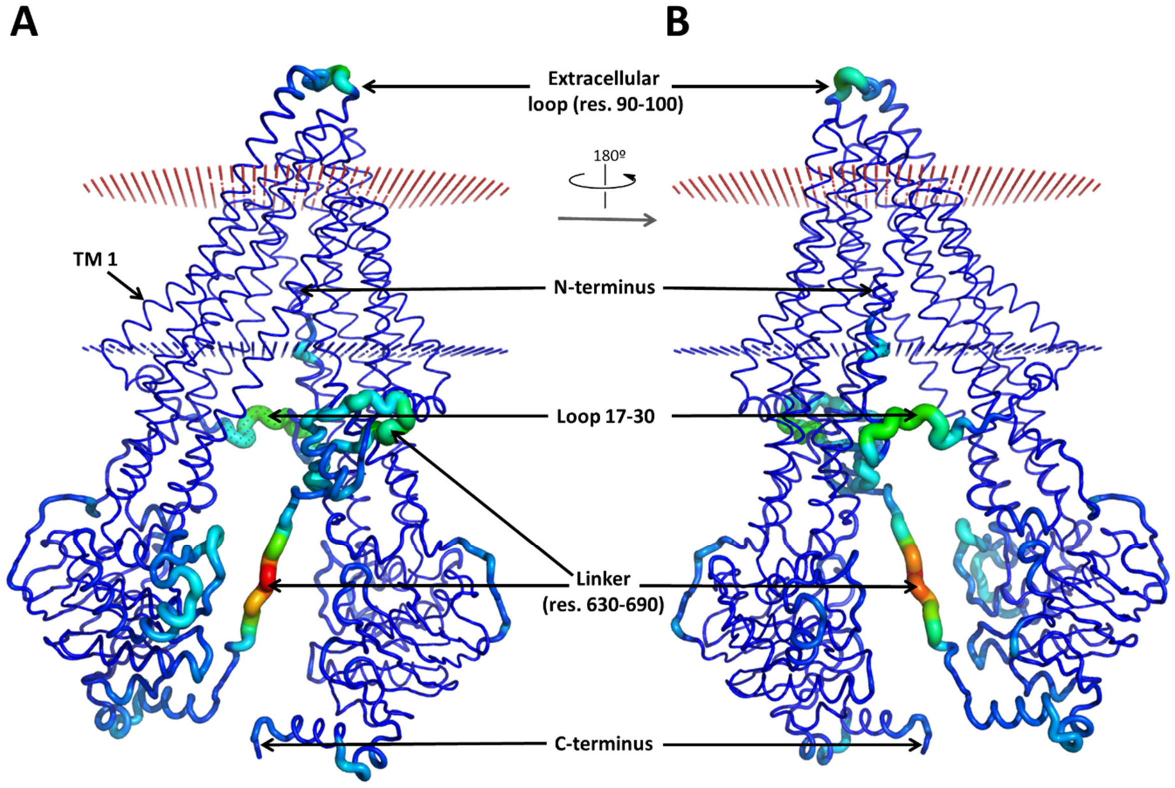

Fig. S4. B-factors obtained from the MD simulation of P-gp. A) Structure of the initial model, and B) after 180° rotation around the y axis. The protein is shown in B-factor putty representation, with the thicker putty and warmer colors corresponding to higher B-factor values; the cell membrane is represented by the blue dots (cytosolic side) and red dots (extracellular side).

2.2 Clustering

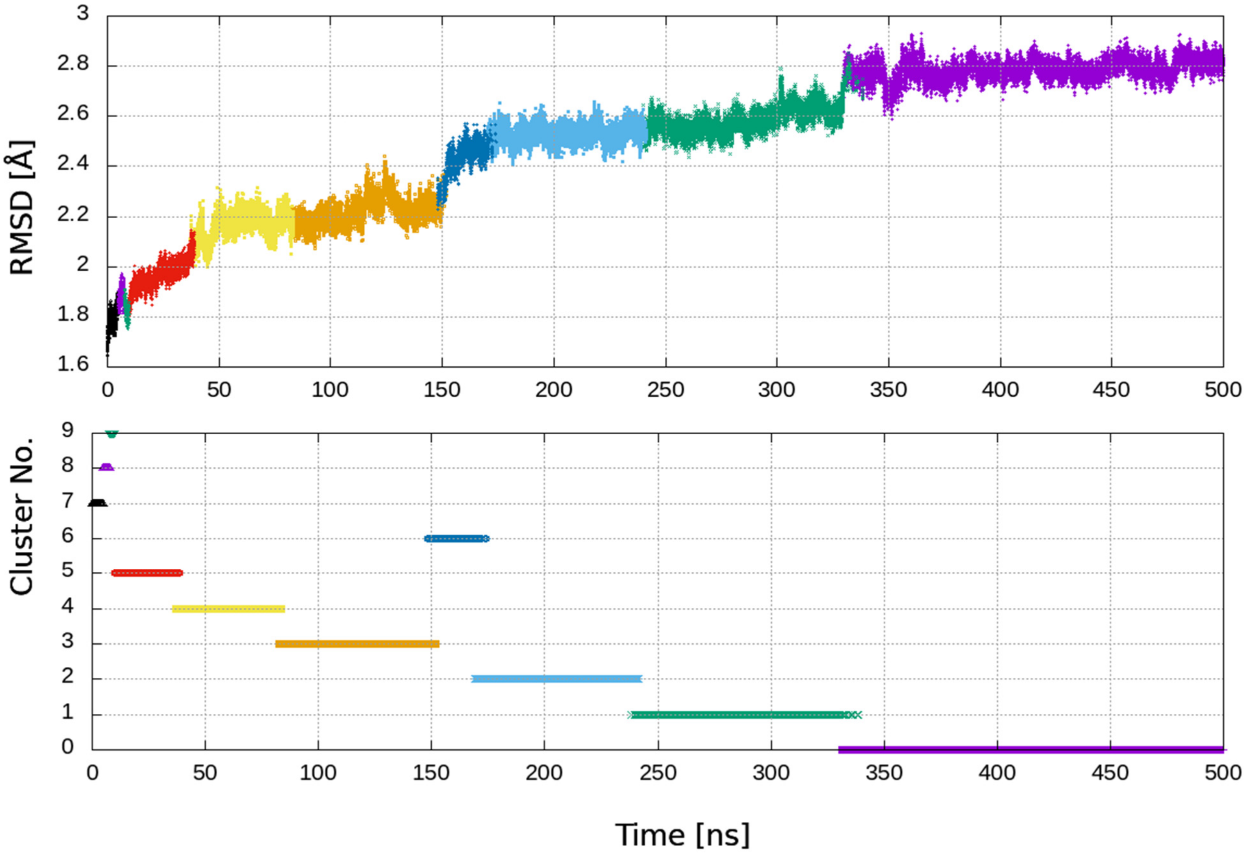

Fig. S5. RMSD and distribution of the clusters obtained from the MD simulations with model 1. Top: variation of the RMSD of the transmembrane residues (for all heavy atoms, as used to define the clusters) with the simulation time, with respect to the initial model 1, colored by cluster; bottom: the clusters' distribution with time during the MD. The population of each cluster decreases with increasing the cluster number (*cluster 0*, in purple, is the most populated).

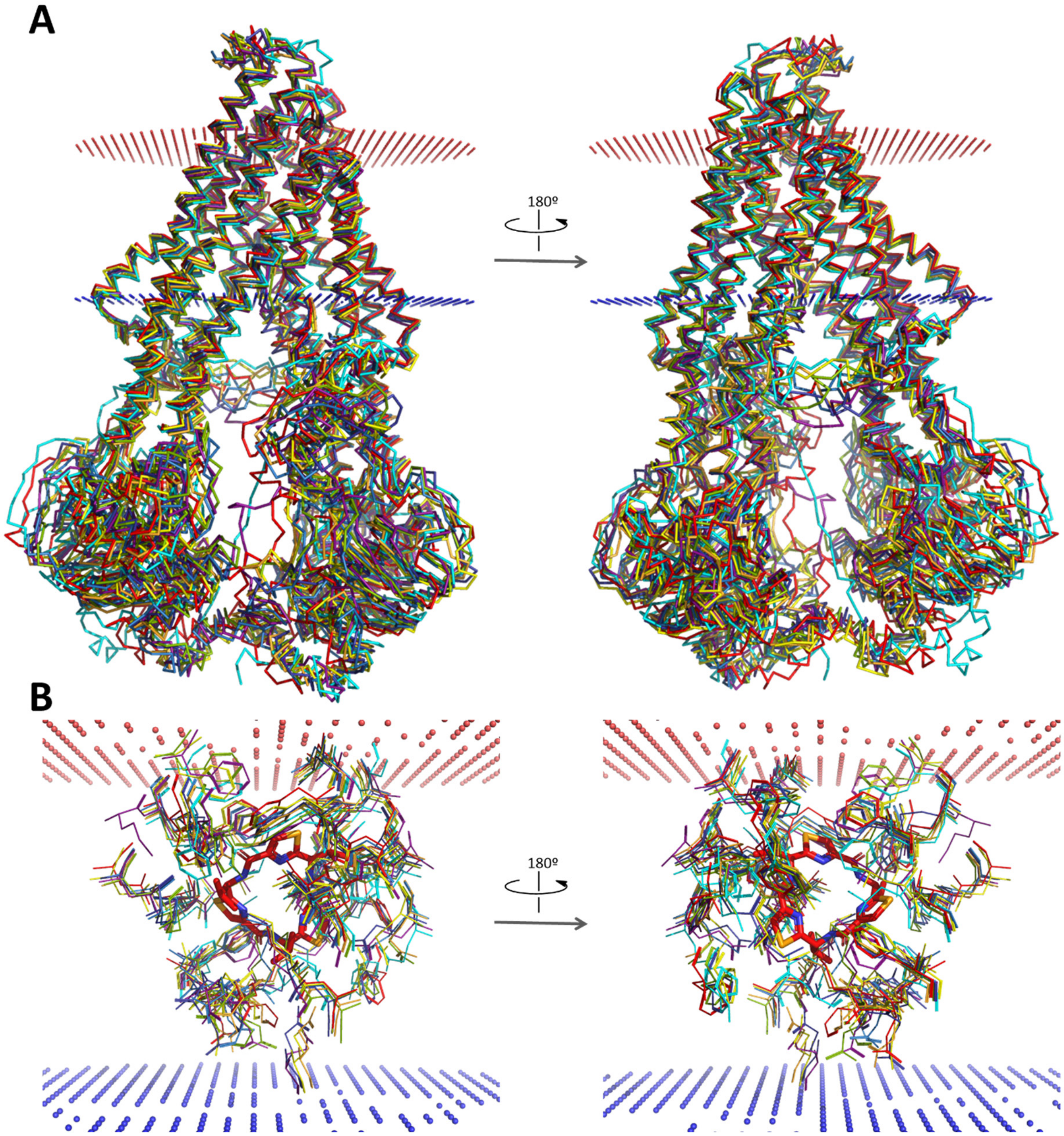

Fig. S6. Superposition of the structures of the top clusters derived from the MD simulation (*cluster 0* to *cluster 6*) with the initial model. A) Global structures in ribbon representation; B) residues at the M site of the drug-binding site (side chains sown as wires), superimposed with the QZ59-*RRR* inhibitor (in red sticks; from a complex with the mouse P-gp, PDB ID 3G60). The initial model is represented in cyan and the clusters in the same colors as the plots in Fig. S4; *cluster 5*, used in the virtual screening, is colored in red; the cell membrane is shown by the blue dots (cytosolic side) and red dots (extracellular side).

Table S4. Quality-assessment of the structures obtained from the MD simulation as predicted by different validation methods.

	**Initial**	**Equil.MD**	***Cluster 0***	***Cluster 1***
ERRAT[c]	92.762	94.360	96.653	95.219
QMEAN[f]	-5.99	-4.11	-3.37	-3.10
MolProbity score[f]	3.05	1.72	1.47	1.54
Ramachandran favoured[f]	84.05%	90.34%	92.45%	92.53%
Ramachandran outliers[f]	6.13%	1.97%	0.84%	0.84%
	Cluster 2	***Cluster 3***	***Cluster 4***	***Cluster 5***
ERRAT[c]	95.638	97.295	94.050	94.676
QMEAN[f]	-3.50	-3.68	-3.29	-3.85
MolProbity score[f]	1.56	1.69	1.65	1.51
Ramachandran favoured[f]	92.95%	91.65%	91.89%	91.69%
Ramachandran outliers[f]	0.84%	1.15%	1.56%	0.76%
	Cluster 6	***Cluster 7***	***Cluster8***	***Cluster 9***
ERRAT[c]	96.970	92.833	94.513	93.519
QMEAN[f]	-3.21	-3.35	-3.24	-3.36
MolProbity score[f]	1.56	1.53	1.67	1.59
Ramachandran favoured[f]	91.95%	91.86%	90.60%	92.20%
Ramachandran outliers[f]	0.92%	1.17%	1.17%	1.34%

[c]calculated with SAVES; [f]calculated with the Swiss-Model Structure Assessment.

3. Molecular docking with the training set

3.1 Evaluation of the docking method

Table S5. Correlations between the scoring results and the biological activities for all docking calculations and re-scoring.[a]

		PEARSON's R_P correlations				SPEARMAN's R_S correlations			
**P-gp variant**	**Structure[b]**	**AutoDock Vina**	**SMINA**	**NNScore**	**RF-Score-VS**	**AutoDock Vina**	**SMINA**	**NNScore**	**RF-Score-VS**
**Human models**	**initial**	-0.1803	0.0373	0.3909	0.2202	-0.1965	0.0637	0.1429	0.0901
	**equil.MD**	-0.1243	-0.0025	-0.1964	-0.0596	-0.1750	-0.0242	-0.2352	-0.1516
	cluster 0	0.1630	0.2098	-0.0083	0.0802	0.1235	0.1429	-0.0330	0.1868
	cluster 1	0.0301	0.2579	0.1869	0.2437	0.0441	0.2835	0.1077	0.1604
	cluster 2	0.0714	0.1913	-0.3315	0.2004	0.0485	0.1253	-0.4110	-0.0286
	cluster 3	0.1554	0.4120	0.4328	0.0121	0.1307	0.3538	0.3890	-0.1253
	cluster 4	-0.0637	0.0653	-0.1851	0.0642	-0.1062	0.0813	-0.2615	0.0901
	cluster 5	-0.0308	0.0808	-0.5448	-0.2009	-0.0850	0.1033	-0.4505	-0.2088
	cluster 6	0.2196	0.4016	0.2379	0.2800	0.3620	0.4022	0.4154	0.2923
	cluster 7	-0.0285	0.0706	-0.1510	-0.0268	-0.1038	0.0022	-0.0374	-0.1209
	cluster 8	-0.1802	-0.1699	-0.3518	0.0057	-0.1938	-0.1385	-0.3451	-0.0418
	cluster 9	0.2438	0.2551	-0.3656	-0.0391	0.2276	0.2879	-0.2308	-0.2527
	AVG[c]	*0.0331*	*0.1577*	*-0.1967*	*0.0527*	*0.0000*	*0.3055*	*-0.2484*	*-0.0110*
**Mouse**	**3G60**	-0.0183	0.1415	0.1717	-0.1418	-0.0509	0.0593	0.0857	-0.5604
	**4M1M**	0.1762	0.1290	-0.3695	-0.0452	0.1942	0.0945	-0.2044	-0.1868
**Human cryo-EM**	**6C0V**	0.0214	0.0877	0.0414	0.0113	0.0664	0.2615	-0.0066	-0.0857
	**6QEE**	0.3314	0.3387	-0.3951	-0.0747	0.2863	0.2571	-0.2659	-0.2571
	**6QEX**	-0.0418	0.0464	-0.2722	0.0480	-0.1149	0.0681	-0.2747	-0.0198

[a]Pearson and Spearman's correlation (R_P and R_S, respectively) between the docking scores and the biological activities of the test set of compounds; [b]*initial* is the structure of model 1 as obtained from I-TASSER, *equil.MD* is the structure after one cycle of equilibration MD, *cluster 0* to *cluster 9* are the clusters discussed above; [c]correlation obtained from averaging the scores of all clusters for each compound. Color coding: darker green for better correlations (more negative) and darker red for worse (more positive).

4. Virtual screening

4.1 Structures of the flavonoid compounds

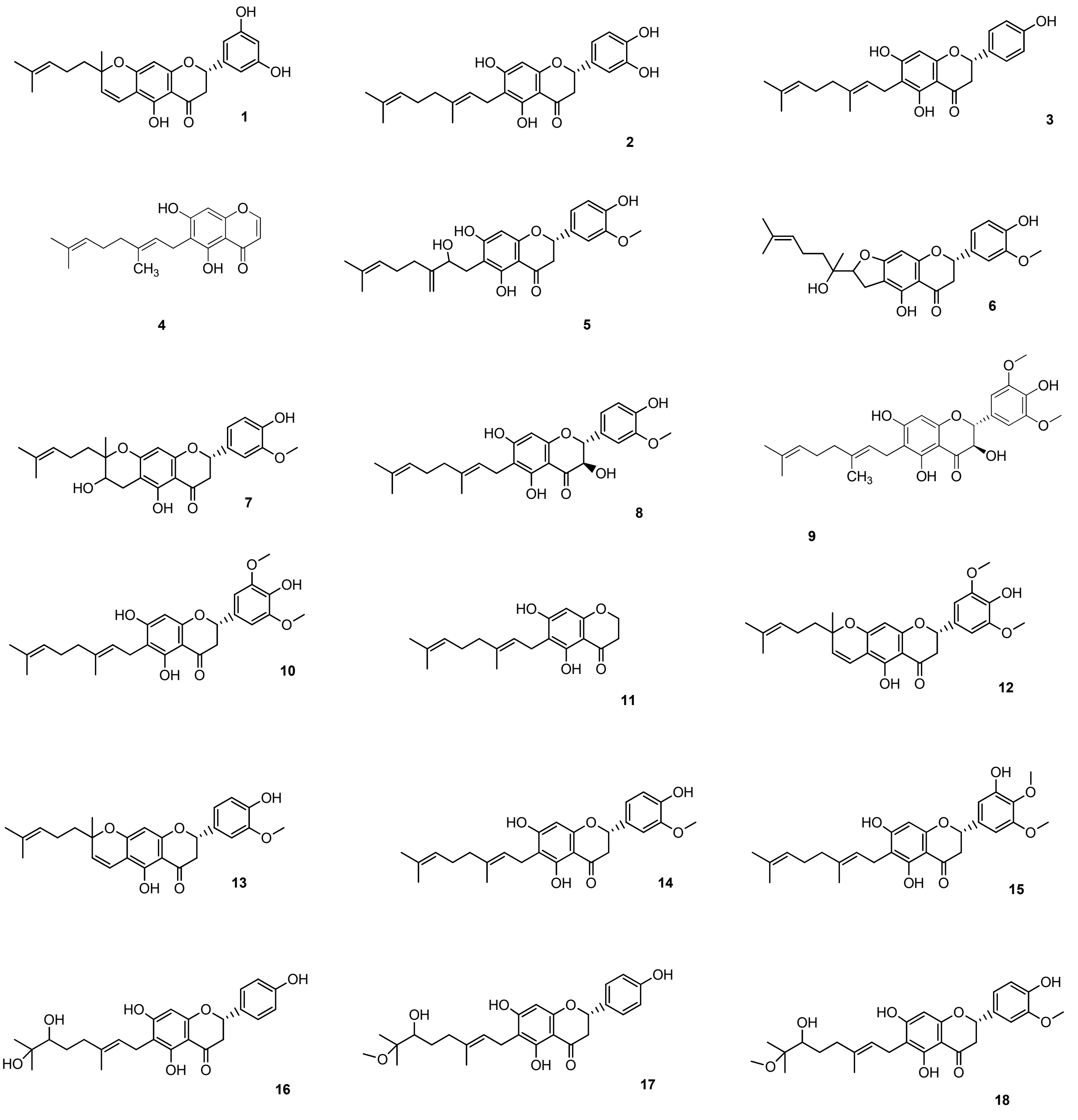

Fig. S7. Structures of the screened flavonoid compounds. The labels represent the sequential numbers in this work.

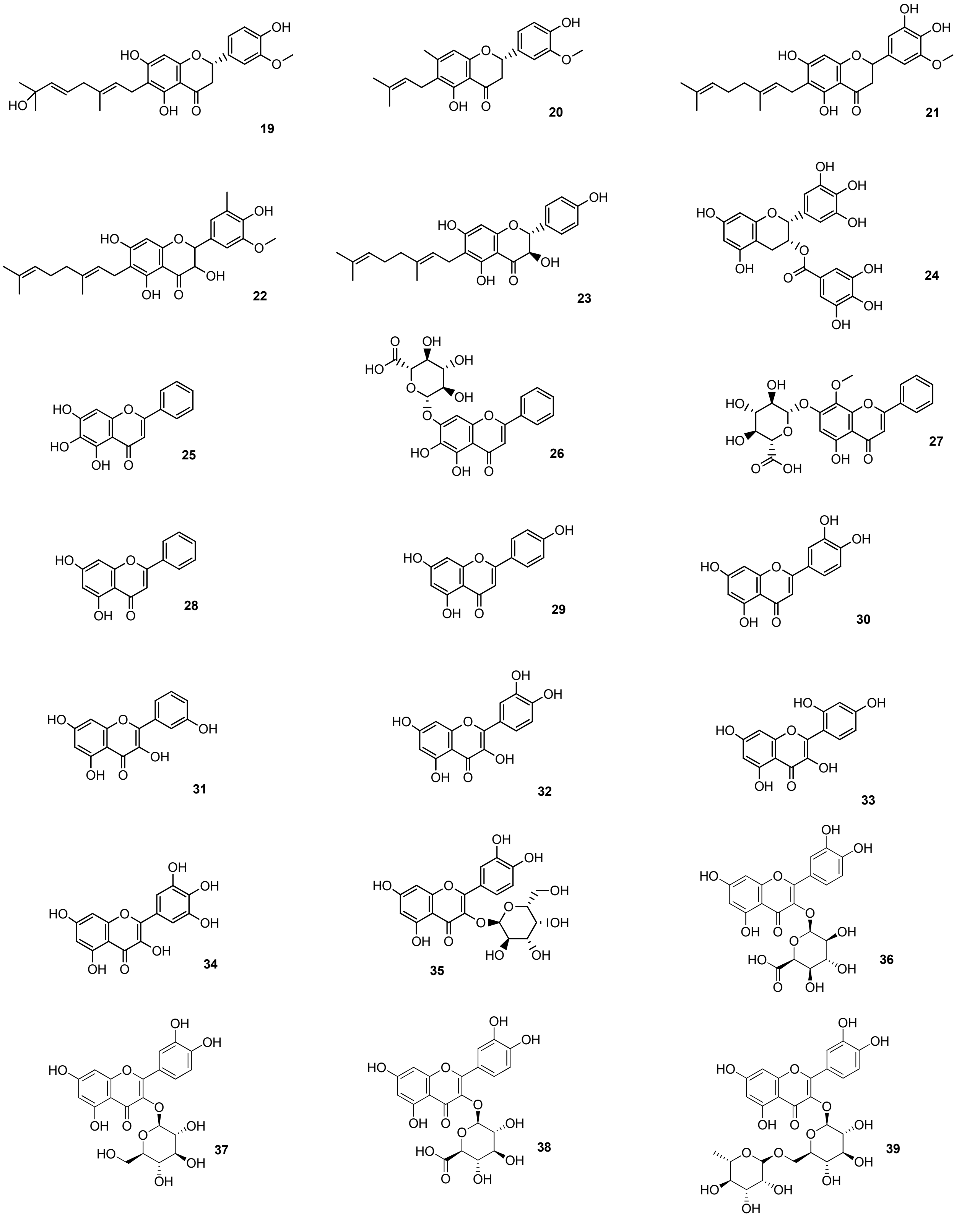

Fig. S7. Structures of the screened flavonoid compounds. The labels represent the sequential numbers in this work. (*cont.*).

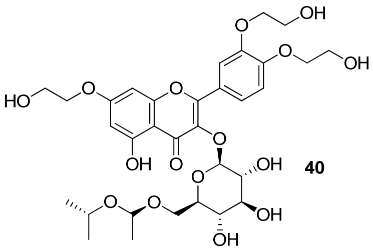
40

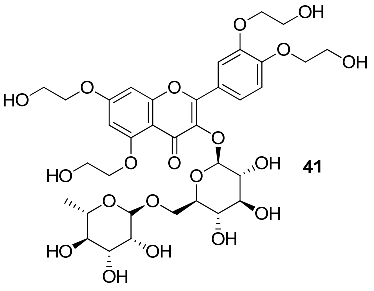
41

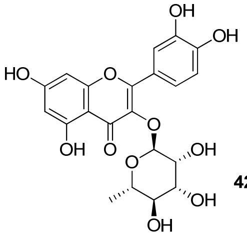
42

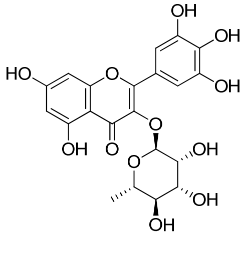
43

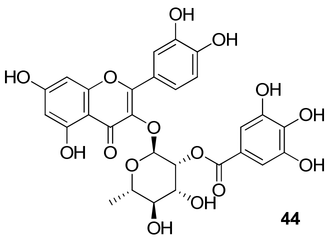
44

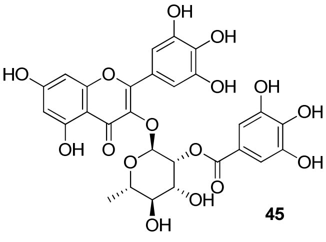
45

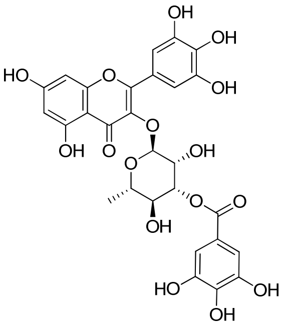
46

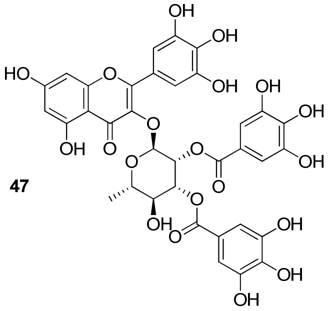
47

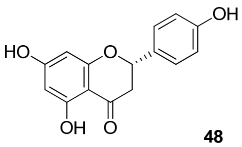
48

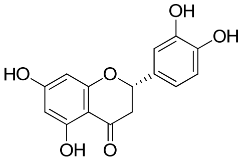
49

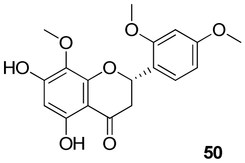
50

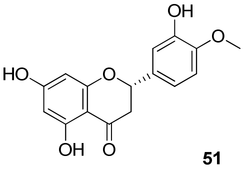
51

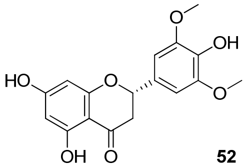
52

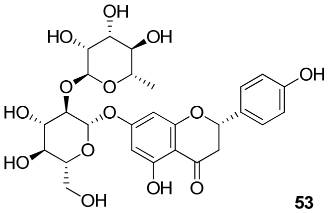
53

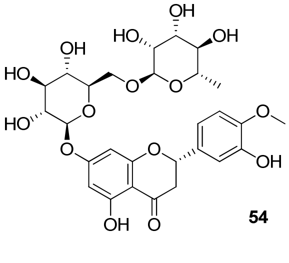
54

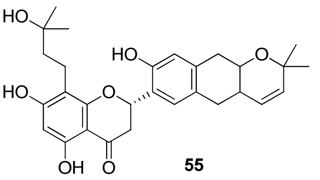
55

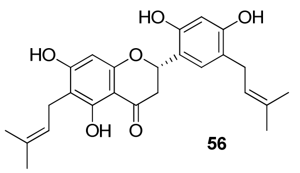
56

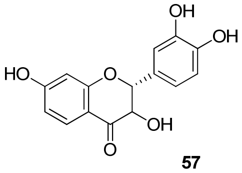
57

Fig. S7. Structures of the screened flavonoid compounds. The labels represent the sequential numbers in this work. (*cont.*).

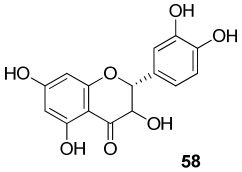

58

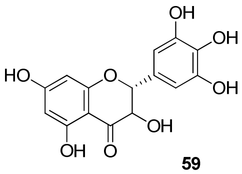

59

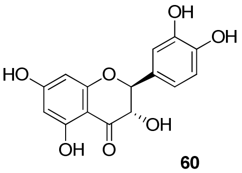

60

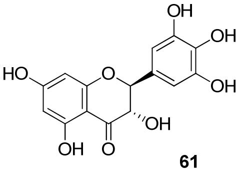

61

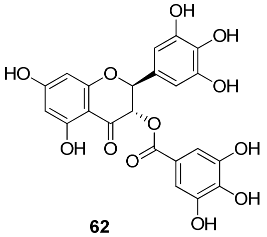

62

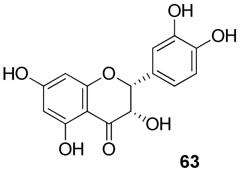

63

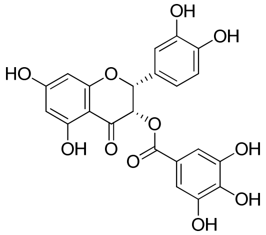

64

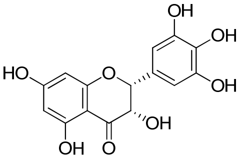

65

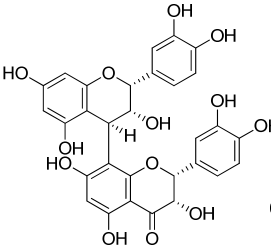

66

67

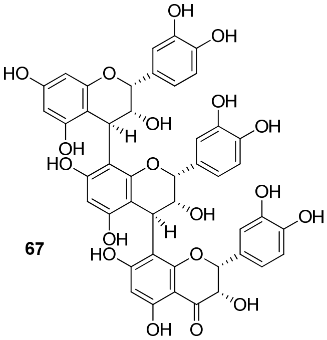

68

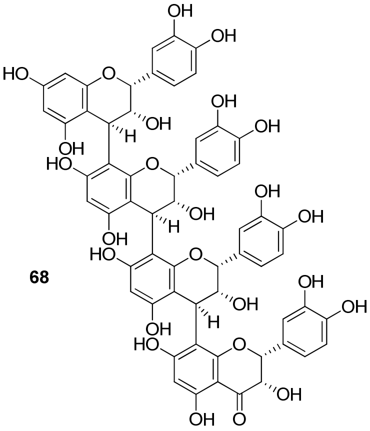

69

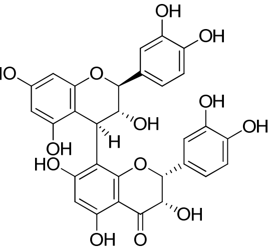

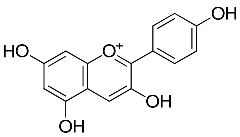

70

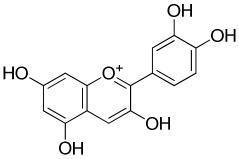

71

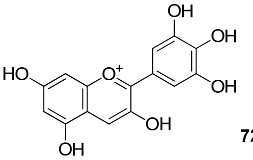

72

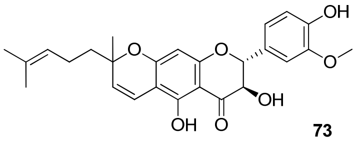

73

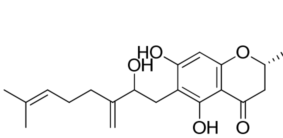

74

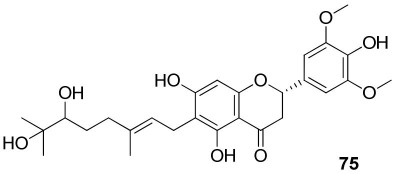

75

Fig. S7. Structures of the screened flavonoid compounds. The labels represent the sequential numbers in this work. (*cont.*).

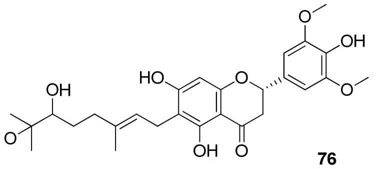

76

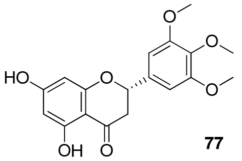

77

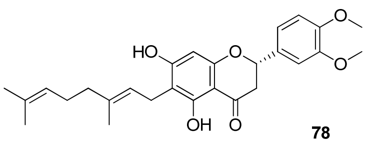

78

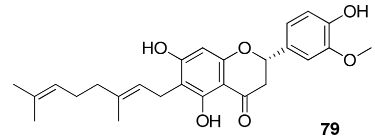

79

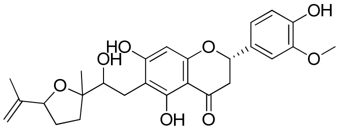

80

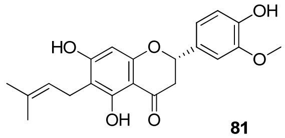

81

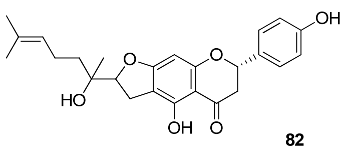

82

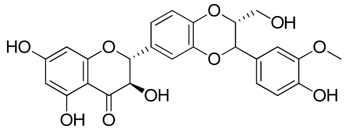

83

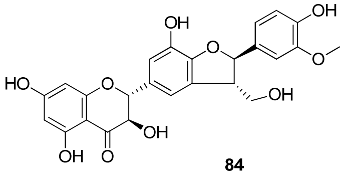

84

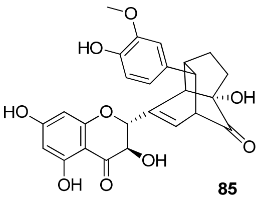

85

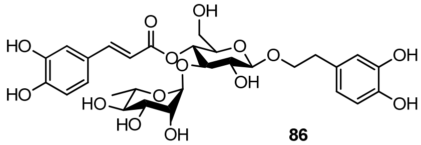

86

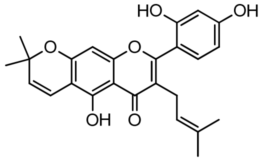

87

Fig. S7. Structures of the screened flavonoid compounds. The labels represent the sequential numbers in this work. (*cont.*).

4.2 *Virtual screening results with the flavonoids*

Table S6. Complete virtual screening results obtained with NNScore (K_d) and AutoDock Vina (ΔG_{bind}) for the tested flavonoids with *cluster 5*. For comparison and potentially further inhibitor identification, we also add the respective best scores of each compounds with *any* of the structure derived from the P-gp model.[a]

Ranking	Scores from *cluster 5*			Best scores from *any* structure		
	Compound	K_d (nM)	ΔG_{bind} (kcal/mol)	Compound	K_d (nM)	ΔG_{bind} (kcal/mol)
Best lignan	**Lig-(+)-4**	17.5	-7.8	**Lig-3**	8.89	-8.9
Worst lignan	**Lig-11**	359.8	-7.3	**Lig-(-)-8**	212.7	-7.6
Verapamil	**(*R*)-vera**	0.74	-7.9	**(*R*)-vera**	0.40	-8.5
	**(*S*)-vera**	0.46	-7.3	**(*S*)-vera**	0.42	-8.5
1	25	1.16	-7.7	32	1.01	-9.1
2	37	1.27	-9.0	84	1.01	-9.9
3	40	1.30	-8.7	86	1.01	-9.7
4	79	1.35	-9.8	54	1.02	-10.2
5	50	1.39	-7.9	45	1.03	-10.2
6	44	1.52	-9.7	46	1.03	-10.2
7	62	1.52	-9.0	36	1.04	-8.6
8	72	1.63	-7.7	27	1.05	-9.5
9	82	2.67	-9.0	30	1.05	-8.9
10	61	2.85	-7.6	35	1.05	-8.8
11	77	3.02	-8.3	58	1.06	-8.5
12	21	3.15	-9.5	53	1.08	-9.8
13	63	3.21	-8.4	13	1.09	-10.1
14	10	3.49	-9.5	49	1.10	-8.4
15	3	3.80	-9.9	60	1.10	-8.2
16	52	4.26	-8.1	3	1.11	-10.0
17	26	4.34	-9.3	28	1.11	-8.8
18	60	4.59	-7.5	31	1.12	-8.8
19	13	4.65	-9.8	50	1.12	-7.9
20	65	5.79	-8.5	24	1.13	-9.8
21	43	6.32	-8.8	61	1.13	-8.3
22	78	7.93	-9.8	52	1.14	-8.1
23	87	8.10	-9.7	59	1.14	-8.8
24	14	8.86	-9.8	25	1.16	-8.7
25	4	9.62	-8.2	40	1.17	-9.1
26	55	10.2	-10.3	66	1.17	-10.2
27	17	10.4	-8.8	48	1.18	-8.6
28	74	11.6	-9.3	29	1.20	-8.6
29	71	12.1	-7.5	42	1.20	-8.9
30	12	12.7	-9.8	82	1.20	-9.9

31	7	14.3	-10.1	81	1.22	-9.0
32	5	15.3	-9.4	37	1.23	-9.2
33	1	16.8	-9.5	7	1.27	-10.1
34	2	17.8	-9.3	47	1.28	-10.6
35	15	18.4	-9.6	64	1.31	-9.9
36	19	19.0	-9.4	39	1.33	-10.0
37	80	20.1	-9.6	6	1.35	-9.9
38	56	21.8	-10.0	62	1.35	-9.7
39	86	22.5	-9.1	79	1.35	-9.8
40	9	26.0	-9.4	72	1.40	-8.2
41	18	28.2	-9.0	34	1.41	-9.1
42	8	28.4	-9.4	83	1.42	-9.9
43	11	32.6	-8.2	18	1.51	-9.0
44	6	36.6	-9.9	44	1.52	-10.0
45	22	37.3	-9.5	26	1.57	-9.6
46	23	38.8	-9.3	71	1.58	-9.3
47	76	39.1	-8.8	74	1.61	-9.6
48	16	40.5	-8.8	63	1.63	-8.4
49	20	48.3	-8.9	33	1.70	-8.6
50	27	57.8	-9.2	38	1.73	-9.4
51	28	69.3	-7.8	41	1.73	-9.1
52	85	75.4	-10.1	65	1.87	-8.5
53	41	77.4	-9.1	57	2.02	-9.0
54	30	87.2	-8.0	4	2.03	-9.0
55	73	96.4	-9.8	22	2.05	-9.9
56	59	99.4	-7.7	75	2.19	-9.1
57	75	113.9	-9.1	2	2.20	-9.9
58	31	118.3	-8.1	70	2.28	-8.9
59	32	120.3	-7.8	19	2.31	-9.4
60	81	134.6	-8.9	10	2.57	-10.3
61	84	135.0	-9.5	78	2.71	-9.8
62	29	160.2	-7.8	68	2.82	-10.6
63	67	163.5	-8.8	77	3.02	-8.3
64	66	183.1	-9.0	17	3.05	-9.2
65	36	202.5	-8.3	21	3.15	-9.6
66	42	203.7	-8.7	43	3.24	-9.0
67	70	225.0	-7.5	15	3.27	-9.6
68	51	261.7	-8.4	9	3.28	-9.6
69	45	281.1	-9.3	85	3.39	-10.1
70	38	294.6	-9.4	16	3.44	-9.4
71	46	312.6	-9.4	51	3.57	-8.5
72	64	348.0	-9.0	87	3.78	-9.9
73	35	360.1	-8.5	80	3.86	-9.8
74	58	390.6	-7.7	8	3.88	-9.6
75	33	448.6	-7.8	14	4.32	-9.8
76	83	454.3	-9.2	23	4.41	-10.1

77	39	475.5	-9.2	69	6.05	-9.2
78	48	618.2	-7.9	67	6.23	-10.7
79	49	669.7	-8.2	73	6.94	-9.9
80	57	673.2	-7.9	55	7.23	-11.4
81	24	682.4	-8.7	56	7.30	-10.4
82	53	687.8	-9.7	5	7.70	-9.4
83	54	747.9	-9.7	12	12.7	-9.8
84	69	826.2	-8.4	1	14.1	-10.1
85	47	944.1	-9.5	11	16.4	-8.9
86	34	980.2	-8.0	76	20.7	-9.0
87	68	990.9	-9.4	20	21.1	-9.3

[a]The compounds are ranked by the affinity scores predicted by NNScore; for comparison, the ΔG_{bind} scores from AutoDock Vina are presented, as well as the lignans with best and worst scores obtained with NNScore for the training set of lignans (*best* and *worst lignan*, respectively), and the scores for the verapamil enantiomers. Dark green highlight the flavonoids with better scores than the *best lignan* in the training set, faded green the compounds scoring between the *best* and the *worst* lignan, and light grey for compounds worse than the *worst lignan*; the compounds in **bold** are those tested experimentally.

4.3 Binding mode of the flavonoids

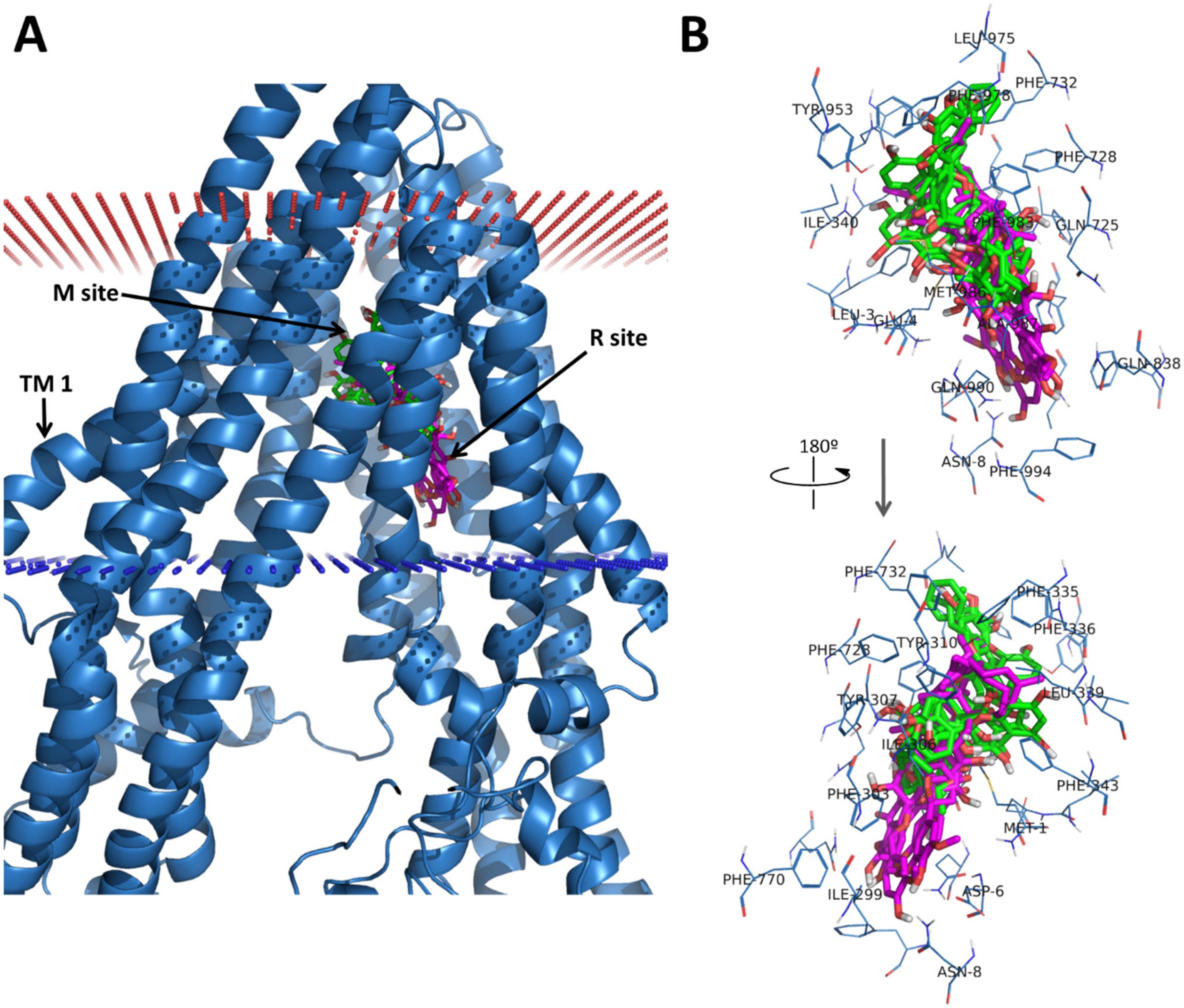

Fig. S8. Molecular docking results of flavonoids **1**–**10** with the human P-gp (*cluster 5* of model 1). A) Overview of the transmembrane binding sites with all flavonoids superimposed (colored sticks); B) detail of the interacting residues. The human P-gp (*cluster 5* form MD) is represented in blue cartoons and lines; the flavonoids are represented as green (binding in the M site) or magenta (binding at both M and R sites) sticks; the membrane is represented by the blue dots (cytosolic side) and red dots (extracellular side).

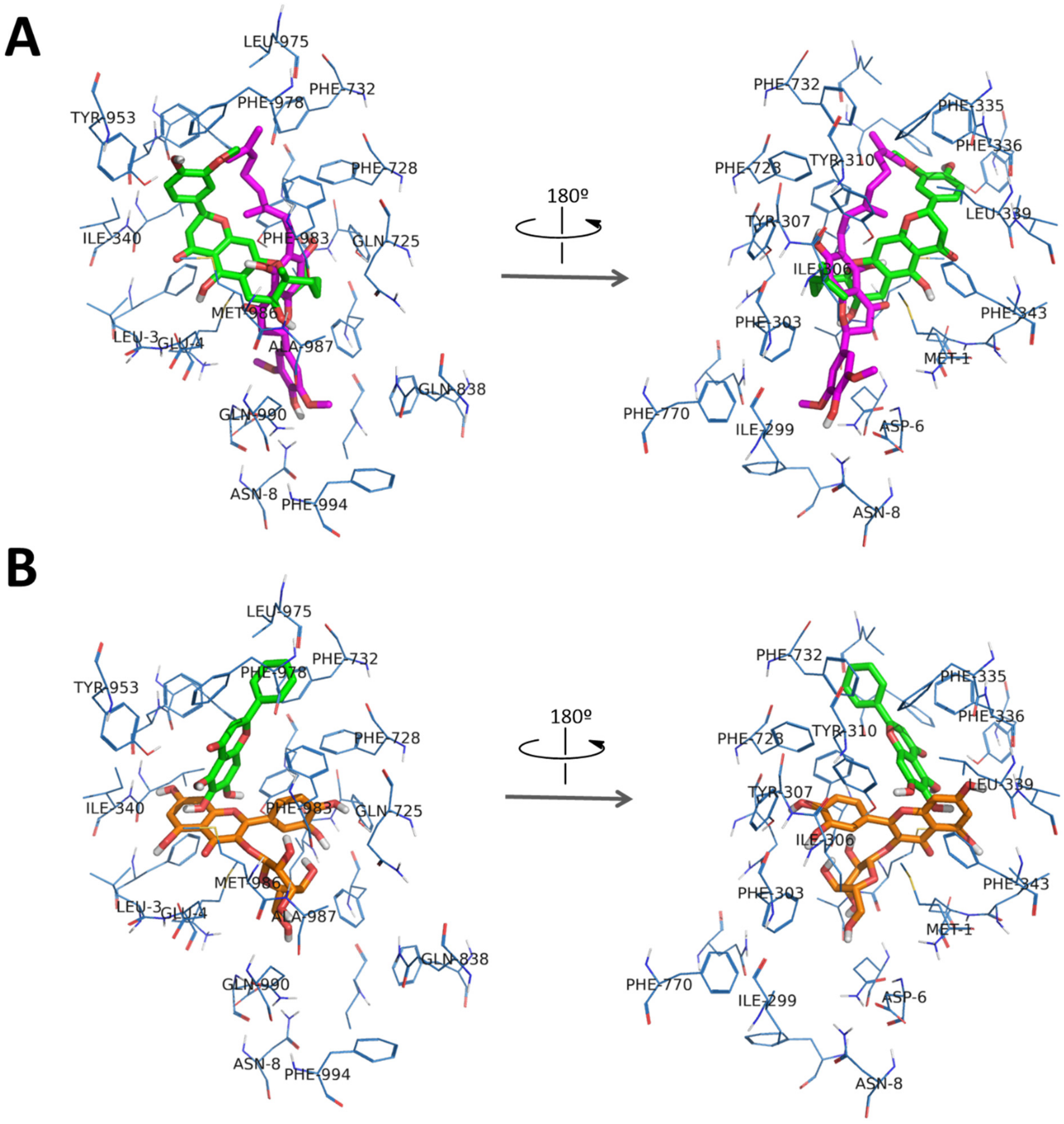

Fig. S9. Molecular docking details for some flavonoids with the human P-gp (*cluster 5* of model 1). A) The biologically active inhibitors **10** (magenta sticks) and **7** (green sticks); B) the top-ranked flavonoids in the virtual screening, 25 (green sticks) and 37 (orange sticks). The interacting residues of P-gp are represented by the blue lines.
